# Dynamical Network Stability Analysis of Multiple Biological Ages Provides a Framework for Understanding the Aging Process

**DOI:** 10.1093/gerona/glae021

**Published:** 2024-01-11

**Authors:** Glen Pridham, Andrew D Rutenberg

**Affiliations:** Department of Physics and Atmospheric Science, Dalhousie University, Halifax, Nova Scotia, Canada; Department of Physics and Atmospheric Science, Dalhousie University, Halifax, Nova Scotia, Canada

**Keywords:** Biological age, Complexity, Eigen analysis, Systems biology

## Abstract

Widespread interest in nondestructive biomarkers of aging has led to a multitude of biological ages that each proffers a “true” health-adjusted individual age. Although each measure provides salient information on the aging process, they are each univariate, in contrast to the “hallmark” and “pillar” theories of aging, which are explicitly multidimensional, multicausal, and multiscale. Fortunately, multiple biological ages can be systematically combined into a multidimensional network representation. The interaction network between these biological ages permits analysis of the multidimensional effects of aging, as well as quantification of causal influences during both natural aging and, potentially, after anti-aging intervention. The behavior of the system as a whole can then be explored using dynamical network stability analysis, which identifies new, efficient biomarkers that quantify long-term resilience scores on the timescale between measurements (years). We demonstrate this approach using a set of 8 biological ages from the longitudinal Swedish Adoption/Twin Study of Aging (SATSA). After extracting an interaction network between these biological ages, we observed that physiological age, a proxy for cardiometabolic health, serves as a central node in the network, implicating it as a key vulnerability for slow, age-related decline. We furthermore show that while the system as a whole is stable, there is a weakly stable direction along which recovery is slow—on the timescale of a human lifespan. This slow direction provides an aging biomarker, which correlates strongly with chronological age and predicts longitudinal decline in health—suggesting that it estimates an important driver of age-related changes.

The continued search for a biomarker of aging that quantifies the effects of natural aging and antiaging interventions (1–3) has resulted in a proliferation of biological ages (BAs) (3), including recent epigenetic clocks (4) (such as Refs (5–8)). Each BA estimates an individual’s health-adjusted effective age, which may differ from their chronological age (CA). Each BA uses a model that converts a battery of measurements into a univariate proxy for health, either using regression on CA (7) or a heuristic mapping into a specific measure of health such as risk of death (5,6). Popular BAs have been extensively validated and are often sensitive to mortality risk (9) and the effects of antiaging interventions (2). Although it is tempting to simply pick the best BA for a particular application, or aggregate BAs using a heuristic approach (10,11), this risks missing the effects of multivariate interactions during the aging process.

Aging is putatively an interacting multivariate, multicausal process (12–15), as has borne out explicitly in computational studies (16–18). This puts aging firmly in the purview of complexity science, where network analysis can be used to account for potentially catastrophic confounding effects due to interactions such as feedbacks between biological variables (12). Such confounding effects could explain the conflicting results emerging from antiaging intervention studies (2,19,20). Learning the underlying network topology can help us to explain these confounding effects, and also to identify vulnerabilities of the biological system. For example, networks with feedback loops may be vulnerable to run-away effects (12), while bottlenecks through high-connectivity nodes may put a network at risk of complete collapse if those vital nodes are damaged (21,22). Networks also provide a useful tool for visualizing and quantifying causal sequences: both during natural aging and, potentially, after interventions.

Fortunately, the complexity of the problem enables an elegant approach for processing and interpreting a network of interacting BAs. Dynamical stability analysis tells us that systems which are mostly stable—such as living organisms—can be understood by the way in which they respond to small perturbations. That is, we look for longitudinal disruptions to homeostasis and subsequent recovery (or lack thereof) (23). The eigen-directions provide a spectrum of fundamental recovery rates (timescales), oriented to completely account for the complex interactions of the network. These rates describe canonical changes so that we can infer long-term behavior by the slowest recovery rates (23,24), which determine system resilience (25). Taking a long time to recover is indicative of weak stability and hence vulnerability to stochastic stressors, whose effects tend to pile up along slow or unstable eigen-directions. Indeed, prior work on health biomarkers showed that across 4 data sets (2 mice and 2 humans), the dominant risk direction for survival or dementia onset was always the first or second least-stable (slowest) eigen-direction (23). Furthermore, a recent deep learning result from mice has shown the existence of an unstable latent variable (26) with properties similar to the frailty index (FI) (20,27)—notably nonlinear growth with age and sensitivity to antiaging intervention. Since salient aging information naturally condenses into the least stable (slowest recovery) eigen-directions, these least stable eigen-directions are the best way to describe the collective aging behavior of a network of BAs.

By using longitudinal data, we can also potentially infer causality within our network. We have already developed a generic model for homeostasis, which estimates a network of interactions between biomarkers together with steady-state behavior (23). Although our model is trained using observational data (28), we expect that interventions that cause small perturbations will behave similarly to the random stresses, which drive observational data—because these represent the random effects of interventions that individuals experience throughout their lives, such as lifestyle changes, medicine, and disease. Previously, we found that including such directed cause → effect links conferred little benefit in reducing the root mean squared error (RMSE) of biomarker trajectories. Here, we revisit this question with a novel quantitative score based on predicting the correct direction of biomarker change. As a result, we obtain a model that predicts both the causal sequence of events occurring during normal aging and a best guess for how interventions will propagate.

We demonstrate that network analysis can leverage the abundance of BAs to answer fundamental questions about what causes aging and about how aging systems are likely to respond to interventions. We apply our approach to longitudinal multivariate BA data, generating a network interactome capable of capturing the coordinated effects of the BAs. By considering BAs from multiple biological scales we can surmise how the effects of aging propagate from DNA to functional decline. Once the network is estimated, we can analyze its eigen-directions to understand how the aggregate effects of multiple BAs affect the organism as time progresses. This yields dominant natural variables, which are the salient features of aging and provide canonical coordinates. We show that the least stable natural variable is an efficient choice for monitoring the aging process. We also show that the interaction network has vulnerabilities, which are consistent with qualitative theories of aging, and is able to describe confounding effects of model interventions.

## Method

### Model

We model an arbitrary dynamical system near a stable (homeostatic) point as


$$\begin{array}{l} \begin{array}{llll} & {\vec{b}}_{n+1}={\vec{b}}_{n}+W\Delta t_{n+1}({\vec{b}}_{n}-{\vec{\mu }}_{n})+{\vec{\varepsilon }}_{n+1}\; & \;{\vec{\varepsilon }}_{n+1}∼N(0,\Sigma |{\Delta t}_{n+1}|)\; & \;\;{\vec{\mu }}_{n}\equiv {\vec{\mu }}_{0}+\Lambda {\vec{x}}_{n} \end{array} \end{array}$$ (1)

where ${\vec{b}}_{n}$ represents an individual’s set of BAs measured at time timepoint $t_{n}$ and $\;\Delta \;t_{n+1}\equiv t_{n+1}-t_{n}$ is the measurement interval. Each individual has a different number of total measurements before leaving the study, $T_{i}$, and hence eqn (1) applies to the $T_{i}-1$ pairs of sequential measurements; dropout and missing data are discussed below and in Supplementary Material. We refer to this model as the Stochastic Finite-difference (SF) model, reflecting its relationship to the Stochastic Process model (29), which is the generalized continuous version of the SF model (23). The model estimates: an equilibrium position, ${\vec{\mu }}_{n}$, a causal, resilience parameter, W, which captures an interacting recovery network; and a noise term, Σ which implicitly includes additional effects not in the model—such as nonlinear effects and fast dynamical changes. The model is only sensitive to changes which occur slower than the timescale set by $\;\Delta \;\;t_{n+1}$, which for this study is approximately 3 years. This means that short-term changes such as due to a flu infection appear in the noise term, Σ. W captures only long-term “resilience,” that is, longitudinal correlations, over the course of years—such as age-related decline. The equilibrium position, ${\vec{\mu }}_{n}$, is allowed to vary linearly with respect to a set of covariates for each individual, ${\vec{x}}_{n}$, through Λ.

The diagonal elements of W permit recovery toward ${\vec{\mu }}_{n}$, whereas the off-diagonals couple values across BAs,


$$\begin{array}{l} \frac{E(b_{jn+1}-b_{jn})}{E(\Delta t_{n+1})}=W_{jj}E(b_{jn}-\mu_{jn})+\sum_{k\neq j}W_{jk}E(b_{kn}-\mu_{kn}) \end{array}$$ (2)

where E(x) represents the expectation value of x. (In deriving eqn (2) we assume that the current BA values, $b_{jn}$, are negligibly correlated with the follow-up time, $\;\Delta \;\;t_{n+1}$.) This means that if—through intervention or natural aging—the values of some of the BAs change, $b_{k}$, then we will see a change in each different $b_{j}$ via $W_{jk}$. The diagonal elements $W_{jj}$ are the marginal recovery rates of $b_{j}$ ignoring all other BAs, whereas the off-diagonal elements $W_{jk}$ allow interactions between different BAs—hastening or ameliorating their decline. Hence, changes to one BA can propagate into the other BAs, allowing for a central driver of either age-related dysfunction or antiaging treatment. The system only stops when each $b_{j}$ simultaneously reaches its equilibrium position, $\mu_{jn}$. As we will see, the estimated equilibrium positions can only be reached well beyond the lifespan of normal humans and hence the system drifts indefinitely. The interactions, W, can be simplified by diagonalization using the eigen-decomposition to yield a set of composite natural aging variables $z_{k}$ which satisfy


$$\begin{array}{l} \frac{E(z_{kn+1}-z_{kn})}{E(\Delta t_{n+1})}=\lambda_{k}E(z_{kn}-{\tilde{\mu }}_{kn}) \end{array}$$ (3)

for $\vec{z}\equiv P^{-1}\vec{b}$ and $\tilde{\vec{\mu }}\equiv P^{-1}\vec{\mu }$, where $\lambda_{k}$ is the associated eigenvalue. We index the $z_{k}$ by their sorted eigenvalue strength, such that $z_{1}$ has the greatest (closest to +∞) eigenvalue $\lambda_{1}$. (For simplicity, we drop the tilde notation for the remainder of the paper.) Observe that the natural aging variables, $z_{k}$, do not interact: they either increase, decrease or stay the same, depending on the value of $Re\left(\lambda_{k}\right)$ as can be seen by iterating eqn (3). ($Im\left(\lambda_{k}\right)\neq 0$ contributes oscillations, but we focus exclusively on the real parts of eigenvalues in the present study.) The timescale over which these changes occur is set by ${\left|Re\left(\lambda_{k}\right)\right|}^{-1}\equiv {\left|\lambda_{k}\right|}^{-1}$, that is, the absolute value of the real part of $\lambda_{k}$ ($\lambda_{k}$ has units of $years^{-1}$). The inverse timescale, $\lambda_{k}$, determines how quickly the average individual reaches a steady-state. Since the $z_{k}$ are independent via eqn (3), events or interventions that modulate only $E\left(z_{k}\right)$ will not affect any other $E\left(z_{j}\right)$. The payoff of this approach is twofold: aging information gets compressed into a few specific variables, and the interconnected system behavior is greatly simplified.

The $z_{k}$ are able to drive the observed BAs through the mapping $P\vec{z}=\vec{b}$, which will spread out the effects across several BAs since P is often dense (23). In general, the slowest $z_{k}$ (greatest $\lambda_{k}$) have the slowest recovery ($\lambda_{k}>0$ never recover); previously, we observed that the key $z_{k}$ driving changes in $\vec{b}$ are always among the slowest (23) (for health biomarkers).

The eigen-decomposition also lets us decompose the network, represented as a matrix of weights W, into a sum of sub (eigen)-networks (matrices),


$$\begin{array}{l} W=\sum_{i}\lambda_{i}P_{.i}⊗P_{i.}^{-1} \end{array}$$ (4)

where $P_{\cdot \;i}$ is both the *i*th column of P and the *i*th eigenvector, and $\vec{x}⊗\vec{x}\equiv \vec{x}{\vec{x}}^{T}\;$ defines the outer product, ⊗. Each eigenvalue, $\lambda_{i}$, has associated with it a subnetwork, $P_{\cdot \;i}⊗P_{i\cdot }^{-1}$. The network is the sum of all subnetworks, weighted by their associated eigenvalues (e.g., Supplementary Figure S2). This permits us to visually analyze the effective network for each $z_{k}$ using their associated eigenvalue-eigenvector pair, $\lambda_{k}$ and $P_{\cdot \;k}⊗P_{k\cdot }^{-1}$.

We estimated Λ, W, and Σ using linear regression as described in Supplementary Material. We also iteratively impute the expected model mean for all missed measurements, as described in Missing Data.

### Data

We use publicly available longitudinal data from Li et al. (9) Their data are derived from the Swedish Adoption/Twin Study of Aging (SATSA), and include: age, sex, and 9 BAs. The population included *N* = 845 individuals (342 males), average age at entry: 63.6 ± 0.3 years (standard deviation: 8.6, min: 44.9, max: 88.0). Individuals were regularly measured with median  Δ t=3 years (interquartile range: 2.3–3.4 years) and a median number of 4 measurements per person (interquartile range: 3–7 measurements, max: 9). Survival data were not included in the data set, and patients were instead labeled as dropouts after their last measurement.

The BAs used in the present analysis are summarized in Table 1. We considered 9 BAs from 4 biological scales: genetic, epigenetic, system, and entire organism. BAs were harmonized to the same scale (~years) as follows: Telomere was multiplied by 69.29 and then we added 20.72 to match the standard deviation and mean of CA. Similarly, Cognition was multiplied by 0.9440 then we added 21.34. We model the dynamics of the 8 BAs (“predictors”) and hold out the FI as a longitudinal health outcome, since it is a good predictor of risk of adverse outcome such as morbidity and mortality (18,30) (the FI is also non-Gaussian (27), in contrast to our model assumptions). We fit a more general network including the FI and CA in Supplementary Material.

**Table 1.** Biological Age Summary (9)

Biological Age	Risk Direction^*^	Scale	Input	Output/Pooling
Frailty index (FI)^†^	Up	Organism	Health deficits^‡^	Mean
Functional aging index (FAI)	Up	Organism	Sensory, grip, pulmonary, and gait^§^	Standardize then average
Cognition	**Down**	System (brain)	Cognitive testing	PC1
Physiological Age (PhysioAge)	Up	System (cardiometabolic)	Biomarkers and physical exam^∥^	PCA then Klemera-Doubal (7)
GrimAge	Up	Epigenetic^¶^	CpGs	Mortality risk^#^
PhenoAge	Up	Epigenetic^**^	CpGs	Mortality risk^††^
Hannum	Up	Epigenetic	CpGs	CA^‡‡^
Horvath	Up	Epigenetic	CpGs	CA^‡‡^
Telomere	**Down**	Genetic	$\frac{Telomere\;length\;}{standard\;deviation}$	—

^*^Direction of change with increasing chronological age.

^†^Reserved as an outcome measure of individual health.

^‡^Score from 0 (none) to 1 (full): disability, disease, and self-reported ill-health.

^§^Self-reported hearing/vision, grip strength, lung strength, and gait speed.

^∥^Male: body mass index, waist-to-height ratio, weight, systolic blood pressure, diastolic blood pressure, hemoglobin, serum glucose (log), and apolipoprotein B. Female: hip circumference, waist circumference, systolic blood pressure, serum glucose (log), and triglycerides (log).

^¶^Trained to emulate smoking pack years and plasma proteins: adrenomedullin, beta-2-microglobulim, cystatin C, GDF-15, leptin, PAI-1, and tissue inhibitor metalloproteinases 1 (6).

^#^Linearly transform mortality risk to match mean/standard deviation of chronological age (6).

^**^Trained to predict time-to-death, which includes a proportional hazard from: albumin, creatinine, serum glucose, C-reactive protein, lymphocytes (%), mean red cell volume, red cell distribution width, alkaline phosphatase, and white blood cell count (5).

^††^Invert 10-year multivariate mortality risk (Gompertz + proportional hazard with 9 covariates) (5).

^‡‡^CA = chronological age.

### Statistics and Data Handling

All analysis and statistics were performed using R version 4.1.1 (31). Errors were estimated by bootstrapping using 100 resamples, unless otherwise specified. All statistical tests are *z*-tests, unless otherwise specified. All error bars are standard errors, unless specified otherwise. Fitting and simulating functions, as well as fitted parameter values, are available on GitHub at https://github.com/GlenPr/stochastic_finite-difference_model.

### Preprocessing

Before fitting, we transformed the BAs at each timepoint using principal component analysis (PCA) where the transformation was learned from the first timepoint (except for the diagonal model). The transformation is isomorphic (information-preserving) (23) so we estimated model parameters in PC-space then mapped them into BA-space. The number of PCs to use was selected by minimizing the 632-corrected RMSE, which was 8 (max/information preserving). 632-correction uses a linear mixture of 63.2% out-of-sample test error and 36.8% in-sample training error (23). We used PCA because selecting fewer PCs than BAs can avoid collinearity—as was done in Supplementary Material when the FI and CA were included in the network. A priori, Telomere was initially batch adjusted using linear regression (32), we observed that Telomere was normally distributed but included a few extreme outliers (right tail). Since these could be artifacts of the batch adjustment, we excluded all outliers with *p* < 10^−5^ (9/6006≪1% of entries).

### Model Selection

For initial model selection, we minimize the RMSE and mean absolute error (MAE). We used 632-corrected error values, since these have minimal bias for our model (23). For ties, we maximize the area under the receiver operator characteristic curve (AUC) (33) of the 8 pooled BAs worsening in the next timestep, which is the probability that the prediction will correctly rank individuals who will see an increase in BA as higher than those who will not (34). We select between a fully flexible W (“FullW”) and 3 simplified versions: the null model (with W=0), diagonal in BA-space (“DiagW”), or diagonal in principal component–space (“SymW,” which has symmetric W).

### Missing Data

Data were missing due to missed measurements and dropout at an overall rate of 76%. We imputed the missed measurements and considered the effect of imputing dropped patients in Supplementary Material—the latter made no visible difference to the final results. Excluding dropout, the majority of (“predictor”) BA values were missing (53%), which broke down as the following missingness: 20% (PhysioAge), 23% (Cognition), 27% (FAI), 60% (Telomere), 74% (Horvath), 74% (Hannum), 74% (PhenoAge), and 74% (GrimAge). The FI was missing in 20% of cases.

Missing data were initially imputed by carrying forward the last measurement, then reversed and carried backwards, then we imputed any remaining missingness using the mean of a multivariate Gaussian independently for each timepoint. This initial imputation was replaced at each fit iteration (×5) by the mean model prediction (expectation-maximization). See Supplementary Material for full details.

Failure to impute could lead to biased conclusions (35) since most missingness in clinical studies is due in part to poor health (36): here we observed that individuals missing all epigenetic BA measurements were significantly older (*p* = 10^−10^, Wilcoxon test). Imputed values for these BAs were higher than observed, ostensibly accounting for this effect. The relatively high missingness makes imputation quality important. Imputation quality was visually assessed as good, with realistic dispersion, trajectories, and age-dependence (Supplementary Figures S3 and S4). The available case analysis had much lower significance levels, but captured most of the coarse-grained features of the imputed analysis (Supplementary Figures S7 and S8). Multiple imputation may give a better estimate of true effect sizes since it accounts for imputation uncertainty, see Supplementary Figure S8; qualitative results were identical to our primary imputation result. We consider only the singly imputed analysis in the main text. All outcome measures consider only observed values.

## Results

We compared several model variants, notably: the full model according to eqn (1) (FullW), the diagonal model eqn (3) either in the PCA basis (SymW) or with the raw BA variables (DiagW), and the null model with W=0. The diagonal elements parameterize self-recovery from perturbations, while the off-diagonal elements parameterize interactions between the variables. The symmetrical W has only undirected links (bidirectional interactions). The RMSE and MAE were worse both for the null model and for the noninteracting model (DiagW)—but did not discriminate the asymmetric W (FullW) from the symmetric (SymW). This indicates that interactions were present and important for prediction (W≠0 and not diagonal). To break the tie between FullW and SymW, we picked the one which best predicted the direction of change in BA at the next timestep (BA went up in 60% of measurements and down in 40%). We found that FullW performed better at predicting this worsening, having both lower MAE at 68% confidence and higher AUC at *p* = .1 using the DeLong test (33) (combined: .04 ≤ *p* ≤ .1). Our final model (FullW) predicted future values with accuracy $R_{train}^{2}\approx R_{test}^{2}=0.65\pm 0.01$, $RMSE_{632}=5.75\pm 0.09\;years$, and worsening AUC of 0.764±0.005.

**Figure 1.**
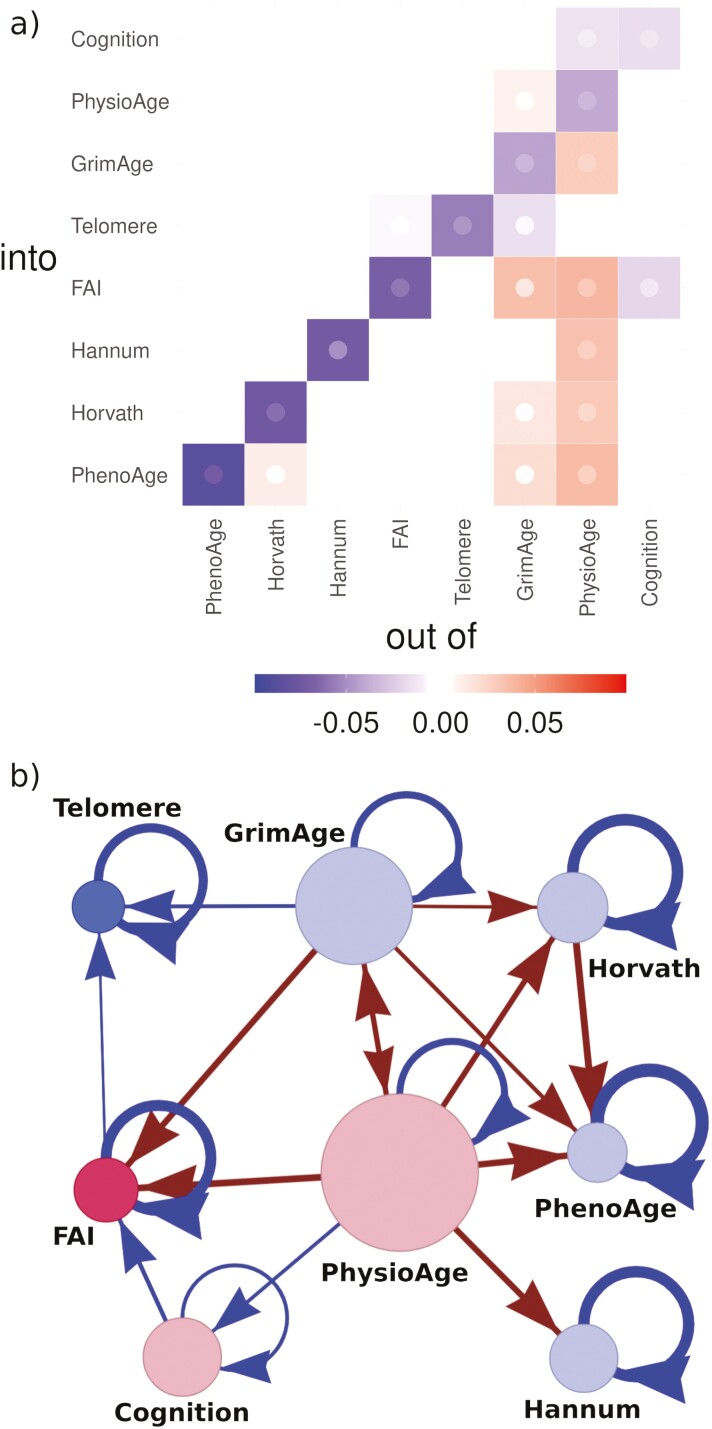
Network interactome. Both representations: matrix (A) and network (B) are equivalent. PhysioAge is the dominant node with the strongest connections, directly driving almost all other BAs (but not Telomere *p* = .3). GrimAge has weaker connections but also has many outgoing connections. All links are significant at *p* < .05. (A) Network weight matrix, W. Our model estimates each interaction parameter in this matrix. Inner point is limit of 95% CI closest to 0: point is most visible for the least significant tiles. Nonsignificant tiles are whited-out (*p* > .05). The elements leaving PhysioAge are typically larger than those leaving GrimAge, and have higher statistical significance (Supplementary Figure S8). Both have weak diagonal recovery $W_{jj}$. Matrix is rank-ordered by diagonal recovery strength. (B) Network representation. Networks encode conditional dependence structures: variables are conditionally dependent if and only if there is a link directly connecting them. For example, GrimAge and Cognition are conditionally independent, since they only interact via an intermediary (PhysioAge). Node size, $n_{k}=\sqrt{\underset{j\neq k}{\sum }W_{jk}^{2}}$ (outgoing strength). Node color indicates biological scale (see Table 1).

The interaction network, W, estimated from the data is presented in Figure 1. W indicates that PhysioAge is the central node and the primary driver of changes over time (the strongest total outgoing links), with GrimAge as an important secondary, high-connectivity node. Observe that there is a positive feedback loop between the highest-connected nodes, PhysioAge → GrimAge → PhysioAge. Explicit inclusion of CA and the FI into the network does not appreciably change the connectivity of these nodes (Supplementary Figure S22); nor does the choice of imputation strategy (Supplementary Figure S7). Note that in Supplementary Material, we confirm that the FI is not connected to Hannum, Horvath, PhenoAge, or GrimAge, as was reported elsewhere using an unrelated statistical model (37). Returning to Figure 1, the high connectivity of PhysioAge would allow it to very quickly propagate dysfunction via eqn (2).

Drift of the BAs with age is the result of the pursuit of equilibrium, $\mu_{j}$. $\mu_{0j}$ ranged from −120 ± 100 to 35 ± 24 years for BAs which decrease with age (Cognition and Telomere) and 127 ± 38 to 190 ± 82 years for the increasing BAs (remaining). (Sex effects were small, ≤10 years; Supplementary Table S1.) In all cases, the equilibrium position is far outside of the age distribution of the population, causing them to drift coherently with age in their respective risk directions (Supplementary Figure S14). In the *z*-picture this effect is concentrated into $z_{1}$ and $z_{2}$ which drift the most, and $z_{3}$ which saturates around age 90—the remaining $z_{k}$ quickly equilibrated and stopped changing with age (Supplementary Figure S13). This is an indication that age-related changes are concentrated into the slowest natural variables, $z_{1}$, $z_{2}$, and $z_{3}$, and primarily into $z_{1}$.

**Figure 2.**
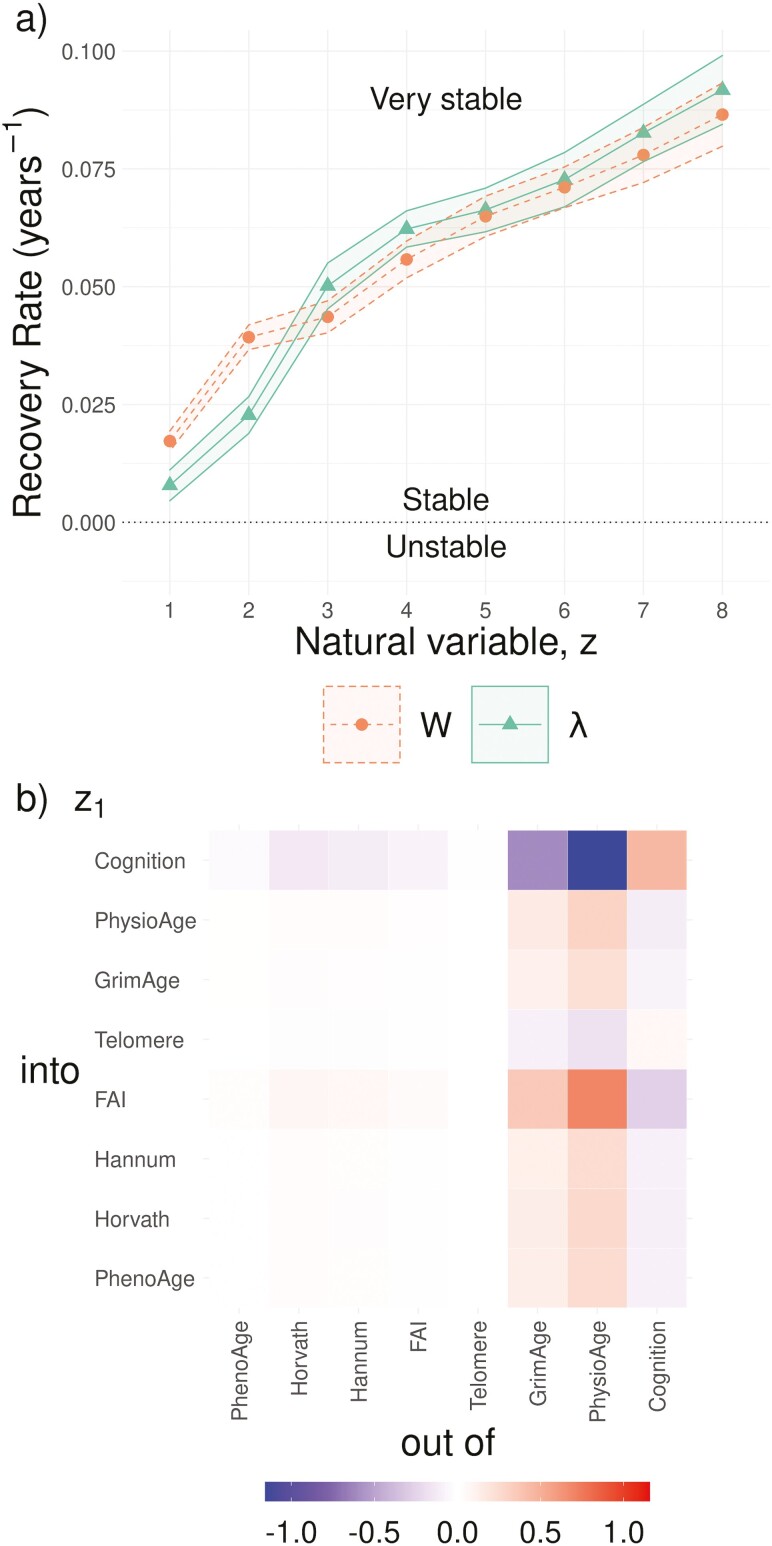
Natural variables of W. Natural variables do not interact, allowing us to analyze their stability. We observed a very weak stability (A), indicative of a slow recovery rate, $\lambda_{1}\approx 0$. The associated eigenvector is visualized in (B), comparing to Figure 1A we see that the slowest eigenvector captures the dense outgoing connections from GrimAge and PhysioAge, including feedback loops. (A) Network stability (resilience). Eigenvalues, $\lambda_{k}$, determine recovery rate, $-\lambda_{k}$. Although the network diagonal ($W_{jj}$) indicates that some biomarkers recover slowly, the network as a whole recovers even slower along $\lambda_{1}$ and $\lambda_{2}$. (Eigenvalue rank is used to index the $z_{k}$.) (B) Eigen-network of $z_{1}$. Associated with each eigenvalue is an eigenvector. The matrix of eigenvectors, P, is used to generate the natural variables as linear combinations of BAs ($\vec{z}=P^{-1}\vec{b}$). The slowest recovering/least stable direction, $z_{1}$, is predominantly PhysioAge, Cognition and GrimAge, all connected into the remaining BAs. Plotted is $P_{1\cdot }⊗P_{1\cdot }^{-1}$, where $P_{1\cdot }$ is the first eigenvector (eqn (4)). Note the role of well-connected BAs with feedback loops: the $z_{1}$ eigen-network has links both above and below the diagonal.

The eigenvalues of W determine system stability, so our focus is on the greatest eigenvalues, which therefore recover slowest (i.e. those closest to zero since they are negative; mean-stability is determined by eqn (3)). The eigenvalues are presented in Figure 2A. Both $z_{1}$ and $z_{2}$ (green triangles) are notably slower than the slowest diagonal elements $W_{11}$ and $W_{22}$ (orange points). The associated timescales are $|\lambda_{1}{|}^{-1}=127\pm 53\;years$ and $|\lambda_{2}{|}^{-1}=44\pm 8\;years$. Observe that both timescales are on the order of a typical human lifespan and are significantly longer than the remaining lifespan of the population, which were all older adults (baseline ages 45–88). The timescales, $|\lambda_{k}{|}^{-1}$ determine how quickly the $z_{k}$ converge to the steady state (eqn (3)). This means that the long-time behavior of the system will depend increasingly on $z_{1}$ and $z_{2}$, which will dominate the mapping into the BAs via $\vec{b}=P\vec{z}$. In Figure 2B we visualize the λ_1_-eigenvector using eqn (4). The λ_1_-eigenvector is centered on a fully-outgoing-connected PhysioAge with feedbacks between GrimAge and Cognition. This means that $z_{1}$ represents the collective action of these 3 BAs driving changes in all 8 BAs. Although these 3 BAs already have the slowest marginal recoveries, $W_{jj}$, the collective action of $z_{1}$ is even slower due to interactions between the BAs.

**Figure 3.**
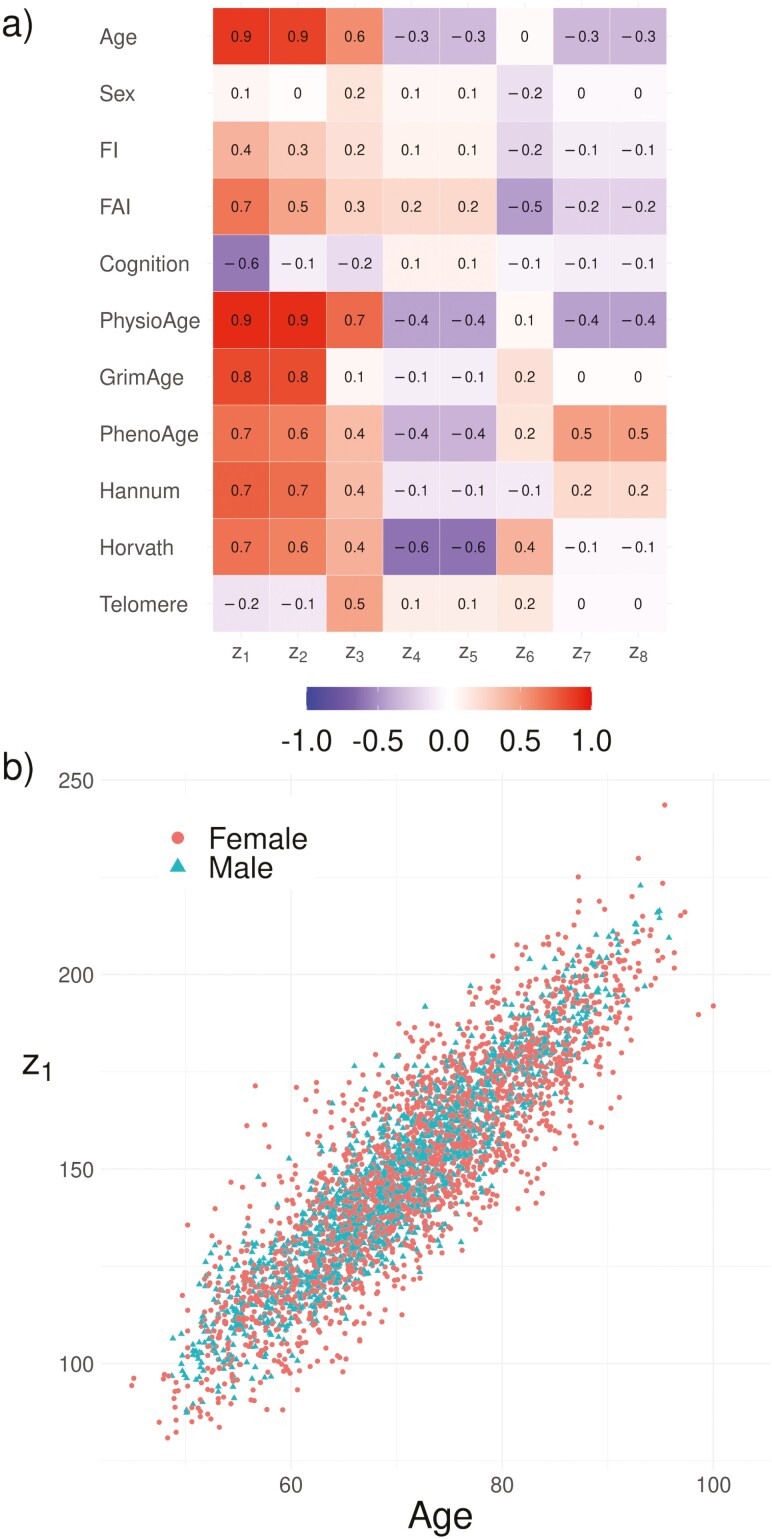
Natural variable correlates. (A) Spearman correlations. $z_{1}$ is strongly correlated with each other BA, CA, and the FI (weakly with Telomere). (B) $z_{1}$ is strongly correlated with CA. As such, $z_{1}$ is capturing essential aging information, including CA and individual health (as estimated by the FI). (Imputed values are included only for the $z_{k}$.)

This bottlenecking of aging information into $z_{1}$ and, to an extent $z_{2}$, is easily confirmed by looking at the correlation matrix, Figure 3A. $z_{1}$ is strongly correlated with almost every BA (weakly with Telomere), and always in the same risk direction. $z_{2}$ shares these correlations except for Cognition, suggesting that splitting between $z_{1}$ and $z_{2}$ is primarily due to differences in cognitive aging rate. Both also had the strongest correlations with CA and the FI of any $z_{k}$. Multivariate ANOVA confirmed that the real part of $z_{1}$ was the dominant predictor of the FI (59% of the explained variance). Correlations with the FI were concentrated into the lowest $z_{k}$, which is clearly demonstrated in the multiply imputed correlations in Supplementary Figure S9 (which accounted for imputation error). In Supplementary Material, we demonstrate that $z_{1}$ and $z_{2}$ are the furthest from equilibrium, which causes them to drift for the entire human lifespan leading to the observed correlation with CA, for example, Figure 3B. Altogether, it appears that the observed age-related changes, including health, are concentrated into the least stable dimensions, particularly $z_{1}$.

Our model, eqn (1), encodes causal dependence of the current timepoint on the previous timepoint. Hence, we can simulate the dynamics after a hypothetical intervention using eqn (1) and the estimated model parameters. We operationalize interventions as an instantaneous rejuvenation of a targeted BA at a specific CA, in a manner which emulates the switching of mortality risk immediately due to an antiaging intervention (38). We simulated matched case and control populations for various interventions, each population contains 50 000 males and 50 000 females and starts at age 60; initial values were sampled from the fully observed BAs of sex-matched individuals in the age range 55–65. We simulated using eqn (1) with timesteps of 1 year. We include a simple model for the FI as a function of the BAs and CA, *R*^2^ = 0.30, to demonstrate how to track the expected change in health as a function of the BAs (details in Supplementary Material).

**Figure 4.**
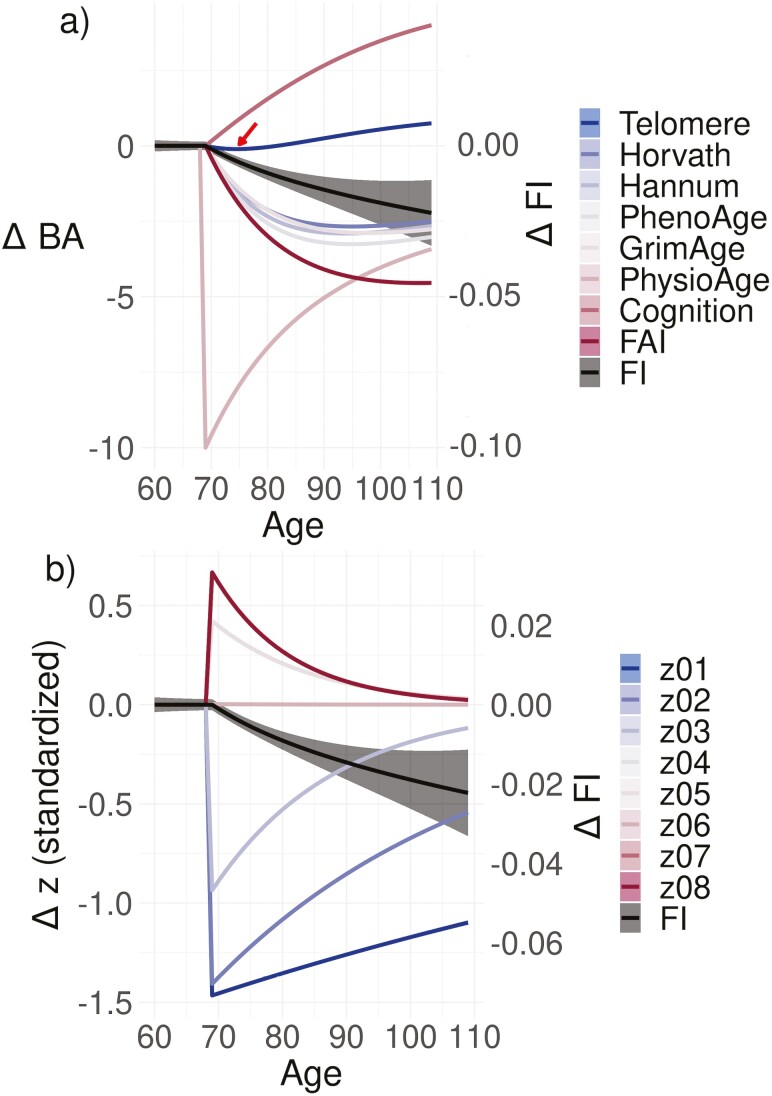
Simulated intervention on PhysioAge. We simulated a hypothetical intervention at age 70, which instantly rejuvenates PhysioAge by 10 years. (A) BA-picture. $\Delta BA\equiv BA_{case}-BA_{control}$. We see an immediate rejuvenation of PhysioAge at age 70 due to the intervention, whereas the remaining BAs have complex delayed effects. For example, Telomere temporarily worsens (arrow) then recovers and ultimately rejuvenates. (B) *z*-picture. $\Delta z\equiv z_{case}-z_{control}$. When working with natural variables, the same intervention effect is immediately spread across all $z_{k}$ which are connected to PhysioAge through $\vec{b}=P\vec{z}$ (P is dense). Each $z_{k}$ simultaneously responds then the $z_{k}$ with fast recovery times quickly revert back leaving only the slow recovery times, $z_{1}$ and $z_{2}$. For the FI, the relative improvement gets better over time since compound (propagated) damage is avoided by the rejuvenation (the FI is unstable). The FI has its own *y* scale as indicated. $z_{k}$ have been standardized for convenient comparison (zero-mean, unit-variance). The sign of $z_{k}$ is arbitrary due to idiosyncrasies of the eigen-decomposition, but could be aligned using their correlation with age or health. Case and control have been perfectly matched for age, sex, and stochastic effects. See Supplementary Material for other simulated interventions. Band is standard error (often smaller than line width).

Having observed the central role of PhysioAge in Figure 1, we simulate the impact of a beneficial intervention administered at age 70, which instantly rejuvenates PhysioAge by 10 years (Figure 4). The intervention causes complex, delayed effects in the other BAs, including an adverse effect: a small, transient telomere shortening (all other BAs improved). This is due to the intervention effect propagating through the network. For example, Telomere worsens (shortens) for about 5 years post-intervention then recovers and ultimately improves after about 10 years post-intervention. Observe that, in contrast to the BAs, the relative FI *continuously improves* with time post-intervention. This is due to the unstable nature of the FI: which grows exponentially with age (27) due to compounding (propagating) secondary damage. Conversely, if we simulate an adverse event, say of disease, which increases PhysioAge by 10 years then we see the same effects with the sign flipped (Supplementary Figure S15). The long-term consequences of the adverse event continue to worsen the relative health (FI) of the case versus control even after the disease. This is consistent with results from a computational network model of disease, which show the long-term FI-effect is due to secondary, compound (“propagated”) damage (39).

It is much easier to understand the effects of the intervention on PhysioAge using the natural variables, $z_{k}$, as shown in Figure 4B. All natural variables, $z_{k}$, connected to PhysioAge immediately improve upon the rejuvenation, then the $z_{k}$ return to normal on the timescale set by $|\lambda_{k}{|}^{-1}$, which means that after 20 years all of the fast $z_{k}$ have mostly recovered. This explains why we should primarily concern ourselves with the greatest eigenvalues, λ_1_ and λ_2_, since their respective natural variables are the only ones with lasting, long-term impacts—good or bad.

Intervening directly on the natural variables, $z_{k}$, gives a greatly simplified picture (Supplementary Figure S16). Because the $z_{k}$ don’t interact with each other, the intervention pinpoints one $z_{k}$ that immediately improves, whereas the other $z_{j}$ are unaffected. Depending on the stability of the intervened $z_{k}$, the effect of the intervention is either gradually lost with age (stable), persists indefinitely (marginal stability/slow dimensions $|\lambda_{k}|∼1\;lifespan^{-1}$), or improves with increasing age (unstable). In the present study, interventions which improve $z_{1}$ are most desirable since they have the strongest relationship with health (Figure 3) and persist for a typical human lifespan. See Supplementary Material for other simulated interventions.

## Discussion

The ongoing proliferation of new BAs (biological ages) presents 2 opportunities for dynamical network analysis: (i) BAs can be used to generate network interactomes to better understand how age-related changes are naturally orchestrated and for comparison to theory, and (ii) there is an increasing need for a robust method of aggregating multiple BAs. Here, we address both opportunities. Using a dynamical model with minimal assumptions, we are able to estimate an interaction network from a collection of BAs. We can also apply eigen-analysis to the network such that we are able to generate dynamically independent aggregate BAs—natural aging variables. The slow recovery and age-related drift of these variables reflect their underlying importance in quantifying the aging process. We propose that such natural variables are the natural language to communicate the aging process, in the same manner that spectral signal analysis has come to dominate many quantitative disciplines.

The estimated interaction network, W, encodes conditional dependencies across BAs of the current timestate from the previous timestate. This permits causal predictions. For example, $W_{ij}>1$ indicates that if $b_{j}$ is lower than $\mu_{j}$ at timepoint $t_{n}$ then it will push down the *i*th variable ($b_{i}$) by time $t_{n+1}$ (eqn (2)). We observed that the $\mu_{j}$ are large enough such that the $b_{j}$ are always pushing each other and are autonomously drifting toward worse health. This drift fills the same role as a “mallostatic” drift with age (23), μ(t), but without explicit inclusion of time. High outgoing-degree nodes, such as PhysioAge and GrimAge, play a key role since they push the other BAs, such that changes to PhysioAge or GrimAge naturally propagate into the other, downstream BAs (e.g., rejuvenation). These relationships are learned from observational changes, rather than interventional (28). However, we know that individuals experience many interventions throughout their lives due to medical interventions, lifestyle changes, and stressors of living, such as disease. This suggests that our observation, W, should be consistent with perturbative (small) interventions. In support, we found that we were better able to predict worsening of BAs by including causal relationships.

Using multiple BAs, we were able to estimate an informative network of interactions, which can enhance our knowledge of the aging process. In the present study, we used BAs of varying biological scales ranging from genetic (Telomere) to whole organism (FAI). We observed that PhysioAge, representing primarily cardiometabolic system changes, was the central node with outgoing arrows directly affecting all other BAs (the Telomere link was not significant). This means that changes to PhysioAge will propagate to the other BAs, eqn (2). This permits PhysioAge to drive the other BAs. We observed a weaker, but similarly well-connected effect emanating from GrimAge including feedbacks with PhysioAge. GrimAge had a strong association with mortality for this data set (similar to the FI) (9), and may represent damage. This implies that cardiometabolic, system-level dysfunction is essential to age-related changes, complemented by genetic/epigenetic damage with feedbacks between the 2 scales. Both PhysioAge and GrimAge had the strongest Spearman correlations with CA at 0.90 and 0.79, respectively (Hannum was third with 0.73). This supports the interpretation that age-related changes emerge first in those 2 variables then propagate outwards, causing the correlation with CA to drop as the information gradually attenuates through the network connections. The key natural variable, $z_{1}$, has a strong association with PhysioAge with important contributions from GrimAge, suggesting that well-connected nodes with feedbacks caused the weak stability, which appears to be primarily related to metabolic functioning.

These observations are consistent with theory. Systems biology informs us that metabolism is a vulnerable point due to its bottleneck (“bow-tie”) through glucose (12,22); its large number of outputs also make it well suited for propagating dysfunction—just as we observed with PhysioAge. Notably, metabolism is considered 1 of 7 “pillar” causes of aging, another is macromolecular damage—which is ostensibly captured by GrimAge (14). The hallmark theory of aging specifically includes epigenetic changes and genomic instability as 2 of 12 “hallmarks”—which GrimAge may be sensitive to—but is considerably less specific toward metabolic changes, grouping cardiometabolic changes into generic changes to intercellular communication (15). This could be an indication that the hallmark theory lacks specificity, although our suite of BAs may be similarly limited. Our suite of available BAs constrains our model to effective dynamics (26), which may differ from the oracle truth—although we can use CA as a catch-all for unaccounted degrees of freedom such as unmeasured BAs (Supplementary Material). Fortunately, BAs are becoming increasingly specific in response to demands for high-dimensional representations of aging (2), such as biological system-specific ages (11). As new BAs emerge, we can continue to use our approach to refine our understanding of the causal relationships underlying aging.

Although the network topology is informative, the dynamical behavior of the system as a whole is obfuscated by its complexity. Eigen-analysis identifies weakly stable, slow-recovery eigenvalues and associated eigenvectors, which can be used to generate aggregate biomarkers, that is, natural variables. The means of natural variables do not interact, giving them simple and intuitive dynamical behavior over time: stable natural variables simply decay toward $\mu_{j}$ on a timescale of $eigenvalue^{-1}$ according to eqn (3). Previously, we observed that across 4 data sets (2 mouse and 2 human) the dominant natural variable for predicting survival or dementia onset was either the first or second slowest eigenvalue, which were always stable but near 0—specifically they took an entire lifespan to recover (23). Ostensibly, these natural variables capture irreversible changes, such as damage. Across studies, we consistently observe that the greatest eigenvalues (slowest; most positive) are associated with the salient features of aging. In the present study these were $z_{1}$ and $z_{2}$ which had the strongest correlations with CA and were associated with health. In contrast to ad hoc approaches to finding the best aggregate BA (10,11), the natural variables are essential features of the dynamical system as a whole. As such, they do not interact and they become increasingly dominant with age, as they store information from stochastic events, making them dominate all of the BAs. The implication is that aging becomes increasingly simple and dependent on these essential natural variables with age (effectively leading to reduced dimensionality (18)). This is a consequence of the mapping $\vec{b}=P\vec{z}$, which leverages redundancies in the BA representation to reduce the dimensionality. This means that aging may be lower dimension that popular theories suggest (14,15). What’s more, the dimensionality of aging may be perturbative in the sense that the first dimension provides the most important information and the subsequent dimensions provide less and less, consistent with previous computational studies (17,18). In general, we see that the long-time dynamical behavior is dominated by the slowest network eigenvalues, representing the directions of slowest recovery and, by implication, the lowest resilience (25).

There is significant interest in using BAs to quantify the effects of antiaging interventions (1,2), with some going so far as to *define* rejuvenation by its prolonged effect on biological age (2). Our simulated interventions highlight the utility of dynamical stability (eigen) analysis in disambiguating network dynamics and identifying optimal intervention targets. Using our estimated model parameters, we were able to simulate a hypothetical intervention at age 70 that instantly rejuvenates PhysioAge by 10 years. Improvement to a BA postintervention has been observed in a number of experiments with epigenetic BAs (2) and other BAs, such as the FI (20). A consistent problem that emerges from these experiments is that health may improve along one dimension at the expense of another, such as increased tumorigenesis postrejuvenation (2), reduced visual acuity following antiaging treatment via metformin (20), and increased frailty following mTORC2 disruption (19)—which is inhibited by rapamycin treatment. Such pleiotropic effects are mediated through some biological interaction network—albeit typically an unknown one.

We observed a similar pleiotropic effect in our simulated intervention, wherein 7/8 of the BAs showed immediate or delayed rejuvenation but worsening was observed in Telomere (shortening) during a 10-year-long transient effect. The general issue is that the interaction network obscures the effects of interventions on a single BA. Such a seemingly simple intervention perturbs the network, which then adjusts all of the BAs through its connections—leading to delayed and unexpected downstream effects. This problem can be avoided by working in the natural variables, $z_{k}$. Because the mapping is linear and invertible, we can easily transform between the BA and *z*-pictures as needed. When working in the natural variables the intervention is greatly simplified: all stable $z_{j}$ revert to the control with a timescale ${\left|\lambda_{j}\right|}^{-1}$, and all unstable $z_{k}$ improve continuously postintervention with timescale ${\left|\lambda_{k}\right|}^{-1}$. This simplifies the problem since we immediately know that ${\left|\lambda_{j}\right|}^{-1}$ is the length of time that a stable $z_{j}$ will differ from control, so we only need to monitor the key natural variables which exhibit high risk and low recovery: $\lambda_{k}≳0$—the remaining $z_{j}$ will quickly forget the perturbation. This requires only that we determine the relevance of each $z_{k}$ to health, which can be done prior to an interventional study (and may naturally compress into the lowest $z_{k}$). Identifying unstable and weakly stable natural variables, and the interventions that modulate them should be a fruitful topic of future research.

An unstable natural variable would be particularly important, although we have now failed to observe an instability in 5 data sets using the SF model, using either BAs or health biomarkers (23). An instability would lead to super-linear growth, such as are observed in the FI (27), dynamical FI (26), and specific plasma proteins (8). Since natural variables drive observed variables via $\vec{b}=P\vec{z}$, unstable natural variable(s) could be of prime importance since all stable natural variables should eventually equilibrate such that all observed age-related changes are driven by the unstable natural variable(s). Furthermore, amelioration of an unstable natural variable would result in continuous life-long improvements such as we saw with the FI in our simulated interventions. However, the vast majority of health biomarkers change linearly with age (40), and BAs are typically designed to track CA in units of time, which, by definition, increases linearly with age. The choice of units used to quantify aging may therefore play a role in determining what super-linear growth means, and therefore stability. A second issue is the effect of a population-level picture, which can mask unstable subpopulations, such as those experiencing or transitioning into chronic disease. This presents an opportunity for more powerful statistical models able to capture such individual effects. Note that a slow instability is indistinguishable from linear drift until advanced ages—where data are sparse. Our current perspective is that organisms live most of their lives in a stable regime of approximately linear decline until a tipping point is reached and nonlinear collapse ensues, quickly leading to organism death or chronic disease.

Although linear drift is reasonable population-level behavior for the BAs and $z_{k}$, a tipping point leading to super-linear behavior is necessary to make sense of terminal decline. Terminal decline occurs in biomarkers of death wherein immediately prior to death they become much worse, driving up the hazard and dropping the survival probability toward 0 over a short period of time. This effect can be seen empirically, for example, in the FI (41), cognition (42), and gait (42), which show a dramatic change in slope occurring around 3–4 years prior to death. Two dynamical phases are needed to capture this effect, such as those elucidated by the saturating-repair model of aging (43,44). In this model, age-related decline begins as a stable, approximately linear system until repair processes “saturate” and a new super-linear phase begins. This model unifies ideas of critical behavior (26,45,46) with damage and repair (47,48), which have been increasingly used within the aging modeling literature. Our prior results from mice and humans support the important predictions made by the saturating-repair model, including “mallostasis” (23): the correlation between mortality hazard and linear drift rate (43). The linear phase is characterized by a linear increase in the homeostatic steady state and is ostensibly driven by asymmetric transitions such as epigenetic methylation and accumulation of disease (49). We hypothesize that the linear phase serves to push individuals toward tolerance thresholds (tipping points) upon which a super-linear phase ensues, quickly leading to death or disease, for example, due to the saturation of repair processes.

It is important to understand that our model is of slow, linear dynamics in W at timescales slower than the interval between measurements (years). Faster dynamics are pushed into the noise term, Σ. The effects of the fast dynamics are to cause biomarkers to rapidly change in values from day to day, whereas the slow dynamics estimated by W are long-term changes over the course of human-equivalent years. The resilience score we estimate using W represents resilience on the timescale of years, which (we believe) is a good timescale to assess aging. Nevertheless, this is in contrast to typical measurements of biological resilience, which are on the short timescale of weeks and shows a clear age-dependence (45). In contrast, we observed no clear age-dependence for the slow-resilience assessed by our W-analysis, noting the large error bars (Supplementary Material). A clear age-dependent drop in the eigenvalues of W could be an indication of saturating repair, but the effect is indirect and bounded at 0 (43,44), which may make it difficult to observe. The correct interpretation of W-resilience is currently unclear. W is capturing the long-term decline due to aging through the longitudinal correlations it causes in the BAs. The smallest eigenvalues have the longest memories and hence they are the natural place for information regarding long term and irreversible changes to build up, making the associated eigenvectors excellent predictors of age-related health.

Our relatively modest predictive performance of worsening (AUC 0.764 ± 0.005) and explained FI variance of approximately 30%, suggest that we have only captured some of the age-related changes. Our model achieved a prediction error on the order of approximately 6 years for BA progression after an average of 3 years of natural aging, representing 65% of the variance. Model error encapsulates the net effect of 4 major sources: missing values, unobserved variables, stochasticity, and model misspecification. The missingness was particularly high in the present study, especially for the epigenetic BAs, which were more likely to be missing for older individuals (thus nonrandom), which could lead to bias—even with good imputation (35). This missingness reduces data quality and quantity, which may explain why we did not achieve statistical significance when comparing only the AUC of FullW versus SymW (*p* = .1). Second, we relied on only 8 BAs to completely predict future health including only sex as a covariate. This limits predictive power and our ability to detect causal relationships because we cannot identify causal connections from unobserved variables (50). Although it is impossible to capture all information, we would hope to find a saturation “elbow” at some larger number of BAs. Third, in addition to intrinsic stochasticity, there is substantial stochasticity owing to nonlab conditions (individual variability) and measurement noise, putting severe limits of predictability. Finally, our model is a local approximation of near-stable homeostasis and does not capture sudden changes, such as may occur due to the emergence of an underlying chronic condition. Although the model could incorporate sudden changes via $\mu_{n}$, this is only possible when they are specified.

There is a trend toward increasingly specific BAs, which are compatible with nonredundant, multivariate representations (11,18). Our approach complements these representations because it provides both causal structure and generates salient aggregate features. Our approach is not limited to BAs, it works for all continuous-valued, longitudinal biomarkers. For example, emerging ‘omics data, such as dynamical changes to proteomics (8) could be a natural target for dynamical network stability analysis. We have previously applied the approach to physiological biomarkers including blood tests, body weight, blood pressure, and other generic health biomarkers (23). Although correlation analysis is the de facto standard, it has a severe shortcoming in that it estimates unconditional relationships, which therefore cannot represent a true network (they do not satisfy the standard axioms of graph theory (51) and hence their interpretation is ambiguous at best). A network link indicates a conditional relationship given all variables in the network, permitting easy and intuitive interpretation. Our approach is very general, and we suggest analysts consider using it any time they apply correlation analysis to longitudinal data. We think that the greatly enhanced interpretability is worth the modest additional computational burden. For more quantitative researchers, our linear model could easily be replaced with a more complex model, such as a deep neural network (17), and the analysis of independent natural variables could remain the same. The key natural variables we observe may also be useful measures for secondary analysis, such as looking for early warning signs for the onset of chronic disease (52).

Our central hypothesis is that BAs provide the raw information needed to generate interaction networks, which can then be analyzed as a whole using dynamical stability (eigen) analysis. Our work highlights the utility of approaches borrowed from complexity science and systems biology. Our results are consistent with aging having several of the key features of complex systems: networks, motifs, and feedbacks, which we show play an important role in understanding age-related changes. This is direct evidence of the importance of complex and systems-level thinking (12) for furthering our understanding of aging. In summation, a simple, interpretable model of the dynamics can be leveraged to estimate the essential effects of aging, and infer the effects of perturbative interventions. We demonstrate that analyzing our fitted data gives results consistent with known theory, making it a potential path forward to operationalizing and testing various qualitative theories of aging. Far from being a curse of plenty, the proliferation of established, new, and increasingly specific BAs may be the key to quantifying and understanding the complex multidimensional changes which characterize the aging process.

## Supplementary Material

glae021_suppl_Supplementary_Material

## Data Availability

Data are publicly available from Li et al. (9). Our fitting and simulating functions using R are available online at https://github.com/GlenPr/stochastic_finite-difference_model. Exact model parameters are provided in CSV files on GitHub.
