# Supplementary material for: Dynamical Network Stability Analysis of Multiple Biological Ages Provides a Framework for Understanding the Aging Process
Source: J Gerontol A Biol Sci Med Sci. 2024 Jan 11;79(10):glae021. doi: 10.1093/gerona/glae021 (PMC11639168; doi:10.1093/gerona/glae021)
Supplement: glae021_suppl_Supplementary_Material [file glae021_suppl_supplementary_material.pdf]

Supplementary Material for "Dynamical network stability analysis of multiple biological ages provides a framework for understanding the aging process"

Glen Pridham[1*], and Andrew D. Rutenberg[1†]

1. Department of Physics and Atmospheric Science, Dalhousie University, Halifax, B3H 4R2, Nova Scotia, Canada

* glen.pridham@dal.ca

†adr@dal.ca (corresponding author)

Summary

This supplemental contains additional information that supports and expands our analysis in the main text. We start with two important utility results: a model for the FI which can be used to transform the FI into a normal random variable, and an extended explanation of the eigen-decomposition of a network matrix into a sum of sub-network matrices. Next we provide additional information on the materials and methods, in particular the biological ages (BAs) used, the imputation process, and important results for estimating our model. The missing data handling section includes reiteration of our key results using alternative methods including multiple imputation and available case. We then provide additional results that supplement the main text. Finally, we end with sensitivity analysis where we consider sex and age–stratified fits, and then the inclusion of additional variables in our network analysis (the FI and chronological age, CA).

Frailty Index (FI) Model

The FI is the average number of health deficits an individual has, which ranges from 0 (perfect health) to 1 (all deficit). We wish to treat the FI like the other BAs but this constrained range causes issues with our model, which assumes normally distributed errors. In this section we seek a transformation of the FI that makes it behave more like a BA: in particular with improved normality. Simultaneously, we seek a model to track FI dynamics during our simulated interventions. In this section we demonstrate that a simple phenomenological model for the FI also naturally yields a transformation that makes the FI approximately normally distributed, solving both issues.

We start by looking for a model for the FI in order to track the simulated FI trajectory. The canonical model is

$$f = f_0 e^{\alpha t} \quad \text{(S1)}$$

where f is the FI and t is the age in years ($\alpha \approx 0.035 \pm 0.001$ years^{-1} and $\ln(f_0) \approx -4.16 \pm 0.001$ are fit parameters[1]). This implies that the FI satisfies the differential equation

$$\frac{df}{dt} = \alpha f \quad \text{(S2)}$$

but unfortunately, the model suffers from the "zero state" problem[1]: if $f = 0$ at any time then it will stay at 0 indefinitely. This is unappealing both conceptually and practically: since $f = 0$ is measured in the present study (32/3162 of FI measurements were 0). The simplest solution to this issue is to introduce a constant damage rate γ such that

$$\frac{df}{dt} = \alpha f + \gamma. \quad \text{(S3)}$$

γ can be thought of as the rate of external damage, whereas α captures the net effect of compound (propagated) damage. This is physically plausible and avoids the zero state problem.

The solution of Eq. (S3) is easily confirmed to be

$$f = f_0 e^{\alpha t} - \frac{\gamma}{\alpha}. \quad \text{(S4)}$$

What about covariates? We have several measures of biological age, $\vec{b}$, which, we assume can be treated as additional information that complements the chronological age, $\alpha t \rightarrow \vec{\beta}^T \vec{b}$ (where $\vec{b}$ includes CA (chronological age)). This yields a log-linear model,

$$\ln(f + \frac{\gamma}{\alpha}) = \vec{\beta}^T \vec{b} + \ln(f_0). \quad \text{(S5)}$$

We selected CA and FAI as $\vec{b}$. We hypothesized then verified that under this transformation the stochastic component of the FI can be approximated as a normal distribution yielding the final model

$$\ln(f + \frac{\gamma}{\alpha}) = \vec{\beta}^T \vec{b} + \ln(f_0) + \xi, \quad \text{(S6)}$$

where $\xi \sim N(0, \sigma')$ is a normal random variable (the model residual). It is known empirically that the FI has a strongly skewed distribution[1] bounded at 0 which will be strictly enforced by the exponential inverse transformation. The transformed FI is plotted against age in Figure S1, along with the model residual, ξ. The interpretation of this transformation is that it converts the FI, f, into a linear function of age with normally-distributed error term i.e. a biological age on its native scale. We expect normally-distributed errors for the population's biological age since the individuals are all over age 40 and hence should have been exposed to many stochastic events that could age or rejuvenate them leading to a normal distribution by the central limit theorem.

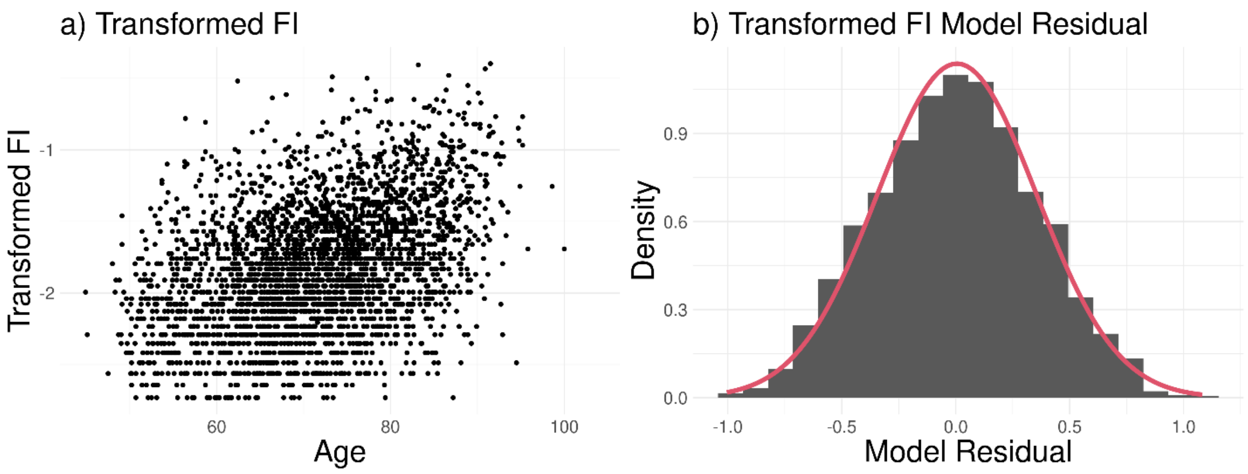

Figure S1: **Transformed FI.** The FI was transformed by $ln(f + 0.065)$ to improve linearity **(a)** and produce an unbiased error term **(b)**. **a)** the transformed FI behaves like a BA having direct proportionality to age, it can be scaled to yield an FI-equivalent BA — which we did in Sensitivity Analysis. **b)** model residual, ξ. The FI is skewed due to being bound at 0, the transformation removes that skewness and instead we have a normally-distributed error term (red line).

Observe that if we can estimate γ/α then we can estimate the remaining terms in Eq. (S6) using linear regression. We iteratively fit a linear model using linear regression and picked the parameter γ/α that minimized the absolute residual skewness. This yielded the estimate $\gamma/\alpha = 0.065$.

Minimizing the skewness is a technical assumption to get a transformed f which is symmetrical, this is needed because the loss functions we use are symmetrical. If the transformed f was asymmetrical then the fitted models would ignore extreme values, instead fitting to the bulk of the distribution. This effect is seen with the untransformed f, for which the linearized models we use ignore individuals near $f = 0$ which leads the models to greatly over-estimate f for healthy individuals (not shown).

In summary, the FI, f, is linearized by the invertible transformation $\ln(f + 0.065)$, allowing us to use linear models in our analysis. Our FI model in the simulations uses the transformation purely as a technical pre-processing step to improve the regression fit. Our network model in Sensitivity Analysis however treats the transformed FI as another BA to include in the model. Readers should be aware that this network model predicts the transformed FI, not the true FI. The transformed FI is an unscaled biological age with slope equal to the damage propagation rate (α). Before fitting to the network in Sensitivity Analysis we scaled to units of age by matching

the mean and standard deviation of CA. The use of the FI as a biological age is discussed by Mitnitski and Rockwood[1].

Eigen-decomposition

Central to our analysis is the eigen-decomposition, which decomposes the network weight matrix, $\boldsymbol{W}$**,** into a set of eigenvalues and respective eigenvectors. The eigen-decomposition of $\boldsymbol{W}$ can be written as

$$\boldsymbol{W} = \sum_{i=1}^{p} \lambda_i P_{\cdot i} \otimes P_{i\cdot}^{-1} \tag{S7}$$

where $\boldsymbol{W}$ has dimensions $p \times p$, $\boldsymbol{P}^{-1}\boldsymbol{W}\boldsymbol{P}$ is diagonal and $P_{\cdot i}$ is the ith column (and eigenvector); note $\vec{x} \otimes \vec{x} = \vec{x}\vec{x}^T$. Hence $\boldsymbol{W}$ is a linear sum of the sub-networks, $P_{\cdot i} \otimes P_{i\cdot}^{-1}$, as shown in Figure S2. Note that the sub-networks, $P_{\cdot i} \otimes P_{i\cdot}^{-1}$, are constrained, rank-1 matrices — this is the proximal cause of their blocky appearance (see e.g. Figure S2a, $P_{\cdot 1} \otimes P_{1\cdot}^{-1}$). The weakly stable eigenvalues have blocky, high-connectivity nodes and feedbacks in their sub-networks because for a sub-network to be strong enough to out-weigh the diagonal in the eigen-decomposition, Eq. (S7), it needs to have a large block of uniform values — which are precisely high-connectivity nodes with feedbacks.

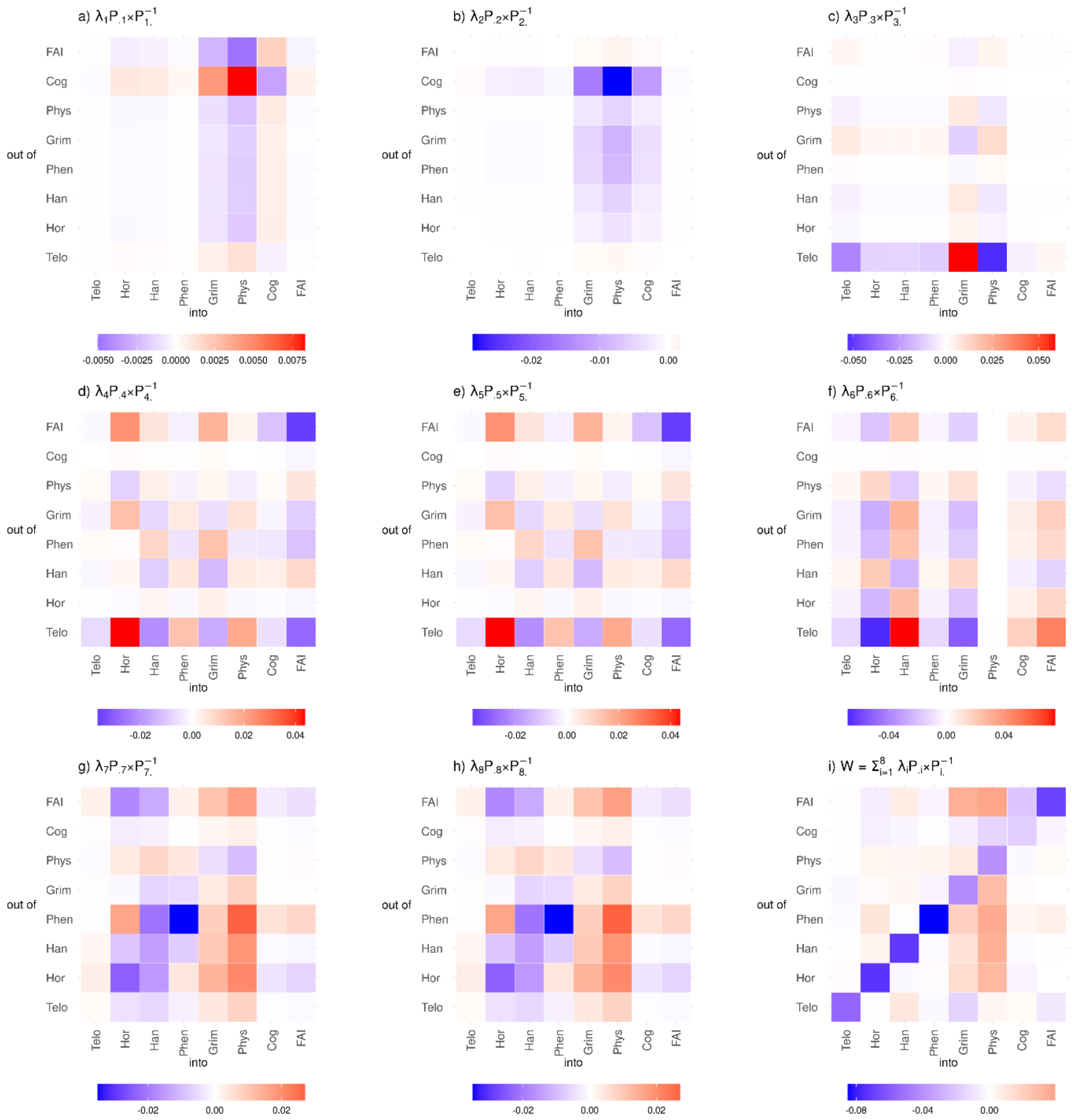

Figure S2: **Network eigen-decomposition**. The network weight matrix, $\boldsymbol{W}$, can be represented as a sum of eigenvalues and sub-networks (proportional to the eigenvectors). The sub-networks ranked by eigenvalue from 1 to 8 are given in **a-h**. **i)** is equivalently the sum of the first 8 and $\boldsymbol{W}$, in accordance with Eq. (S7). Observe the high-connectivity blocks in **a)** and **b)** are associated with low stability. Having links above and below diagonal indicates feedbacks (bi-directional links). Only real components are shown; **d/e** and **g/h** are repeated eigenvectors which differ only in their imaginary components. Causal links flow from "out of" to "into", where the sign of the causal effect is indicated by the colour (red is positive effect, while blue is negative).

Materials and Methods

Here we provide some additional details on the dataset and data handling, starting with a description of the BAs used.

Telomere length is leukocyte T/S-ratio (qPCR assay product over reference)[2]. Batch adjustments of telomere length were made by the original authors using linear regression[2], which justified our choice to exclude extreme outliers, as described in the main text. Horvath and Hannum both capture age-associated epigenetic changes via penalized regression, irrespective of their relationship to health[3]. GrimAge and PhenoAge are more refined since they capture effective survival risk age. GrimAge uses an intermediate step such that only epigenetic changes associated with smoker pack-years or specific proteins are used, the latter are associated with a variety of conditions including inflammation, cardiometabolic dysfunction and cellular functioning[4]. PhenoAge, in contrast, uses epigenetic changes to predict individualized mortality risk using a Gompertz model of survival with proportional hazards[5]. PhysioAge estimates a latent (unobservable) variable which captures mutual age-dependence between the variables and chronological age, each biomarker and chronological age are modelled as a linear function of the latent variable[6]. The variables used are overwhelmingly cardiometabolic, hence PhysioAge likely represents age-related changes to the cardiometabolic system. Cognition is a general cognitive ability score: the first principal component from a battery of cognitive tests[7]. FAI is based on self-reported sensory ability (hearing and seeing), and measured: lung strength, grip strength and gait speed[8]. The FI included 42 measures of health, including self-reported health and sensory, diseases, symptoms, activities of daily living (ADLs) and instrumental ADLs[9] (ADLs are needed to maintain an individual's basic needs; instrumental ADLs are more complex activities needed to live independently[10]).

Missing Data

The majority of ("predictor") BA values were missing (53%), which broke down as the following missingness: 20% (PhysioAge), 23% (Cognition), 27% (FAI), 60% (Telomere), 74% (Horvath), 74% (Hannum), 74% (PhenoAge), and 74% (GrimAge). The FI was missing in 20% of cases.

Prior to fitting, missing data were initially imputed as follows:

1. Carry forward value from previous timepoint to next timepoint,
2. if still missing, carry backwards value from next timepoint to previous timepoint (missingness after steps 1 and 2: 1% (PhysioAge and Cognition), 3% (FAI), 10% (Telomere), 41% (Horvath, Hannum, PhenoAge and GrimAge)),
3. if still missing, impute the time-independent conditional Gaussian mean[11] using other measurements at that same timepoint (missingness after steps 1-3: 0%). (The BAs in this dataset are known to be strongly correlated through their mutual CA-dependence[3].)

After the initial imputation, all of the imputed values were re-imputed using the model mean at each iteration of fitting algorithm (×5).[12] Specifically, we first carried back the current value of the second timepoint estimate into the first timepoint (to avoid inverting $\mathbf{W}$, which has small determinant), then we forward imputed all timepoints past the first using the model mean.

We assessed imputation quality visually both at the population level and for randomly sampled individuals. The population level imputation looked good (Figure S3): the age-dependence and dispersion are similar between both the imputed and observed values. The epigenetic ages are observably high in the mean for most ages, but this is expected because we know that the sub-population who were measured were younger ($p = 10^{-10}$, Wilcox test). We expect the true missing values to be higher than the observed, consistent with what was imputed.

The individual level imputation also looked reasonable, for example Figure S4 shows 10 randomly sampled individuals. We see that the individual trajectories follow the overall population trend while generally interpolating smoothly between observed timepoints (keeping in mind that the biomarkers have multivariate dependencies). We deemed the overall imputation quality was good.

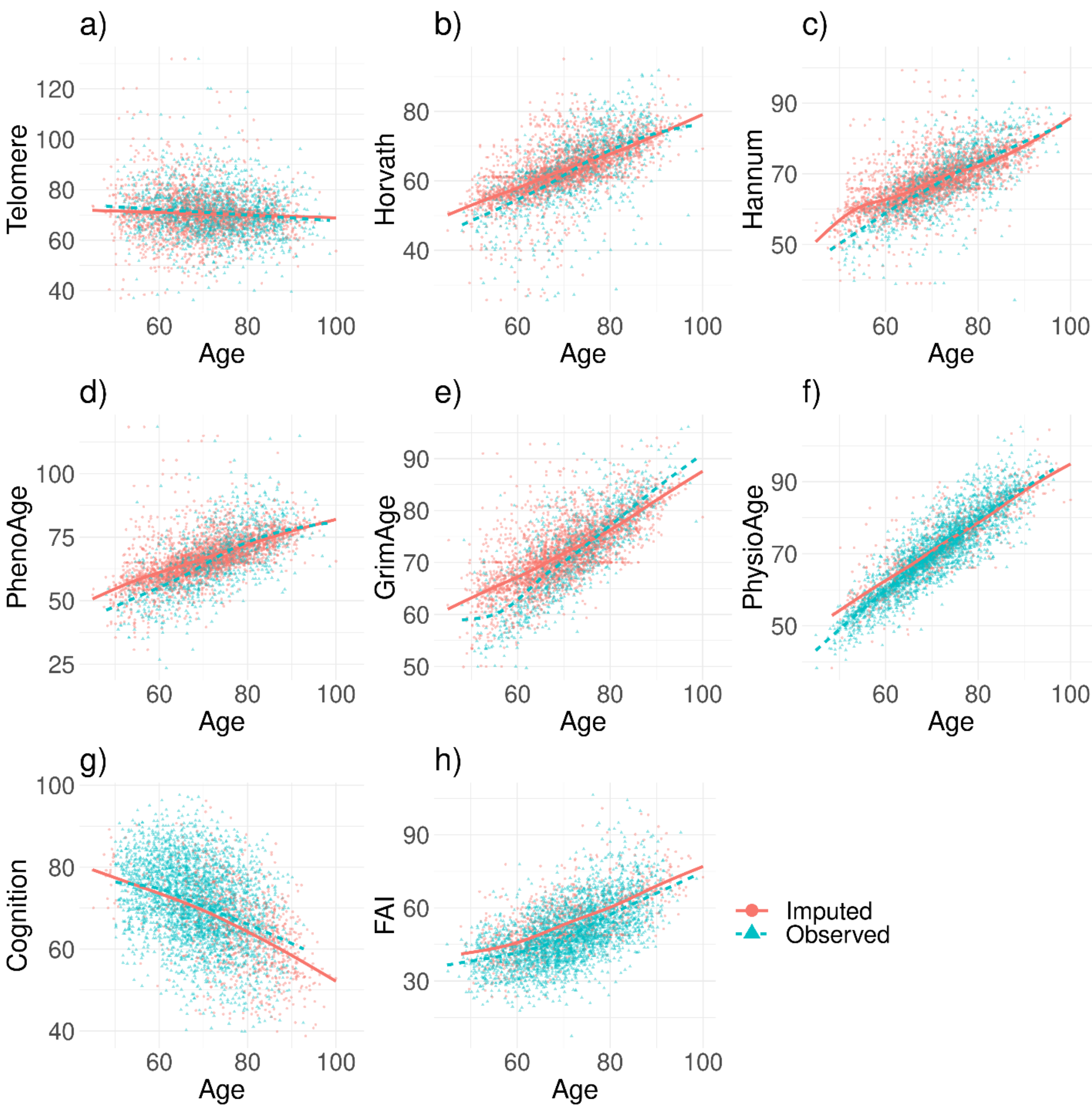

Figure S3: **Imputation quality — age-distribution**. Imputed values (red points) have nearly identical distributions to the observed values (blue triangles): similar dispersion, mean, and age-dependence. The epigenetic ages do, however, show that the majority of imputed values are larger than the observed, this is expected since the individuals missing those values tended to be older (Horvath, Hannum, PhenoAge and GrimAge). Altogether these observations indicated a good imputation. Lines are best fits from a cubic spline additive model using the MGCV package with default degrees of freedom[13].

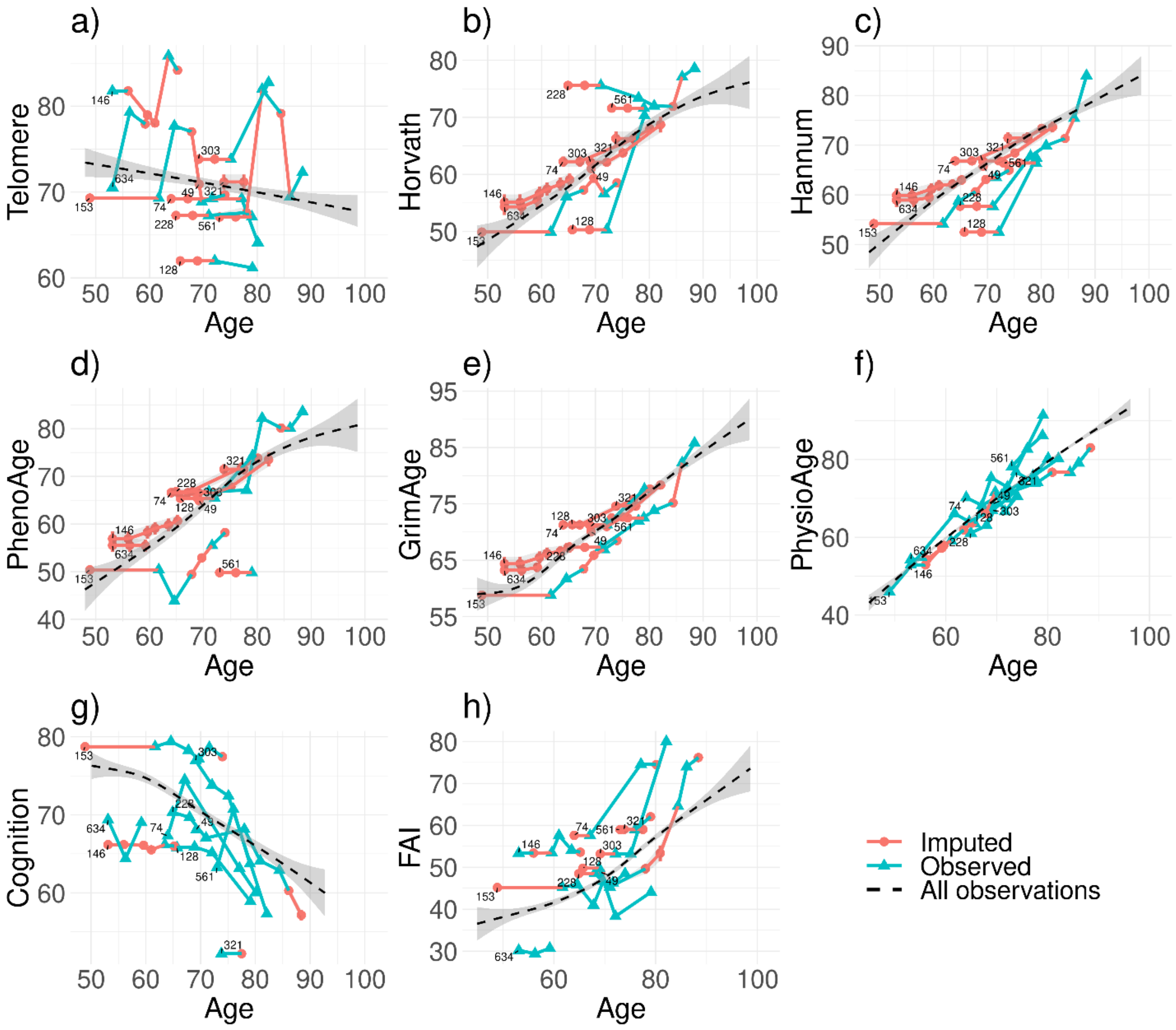

Figure S4: **Imputation quality —sample trajectories.** Sample trajectories for a random subset of 10 individuals (numbered labels). Timepoints which were measured are represented as blue triangles, imputed values are red points (with error bars). The small error bars demonstrate a low sensitivity to parameter estimates. The overall population mean trajectory is included for comparison (dashed line with error band). Interpolated points look good (e.g. PhenoAge for individual 153 at age 70). Overall trajectories look reasonable and follow the overall population trend. Overall population trend is best fits from a cubic spline additive model using the MGCV package with default degrees of freedom[13]. Error bars of imputed values are bootstrap standard deviation.

We investigate the possibility that the imputation strategy could lead to an over or under–estimate of the interaction strengths. In Figure S5 we compare the missingness to the estimated network. We see no evidence of imputation-induced bias. In Sensitivity Analysis we observed that GrimAge and PhysioAge connections were consistent across age strata, which further

supports insensitivity to imputation, since there is a strong age-dependence to the missingness (Figure S19 and S20).

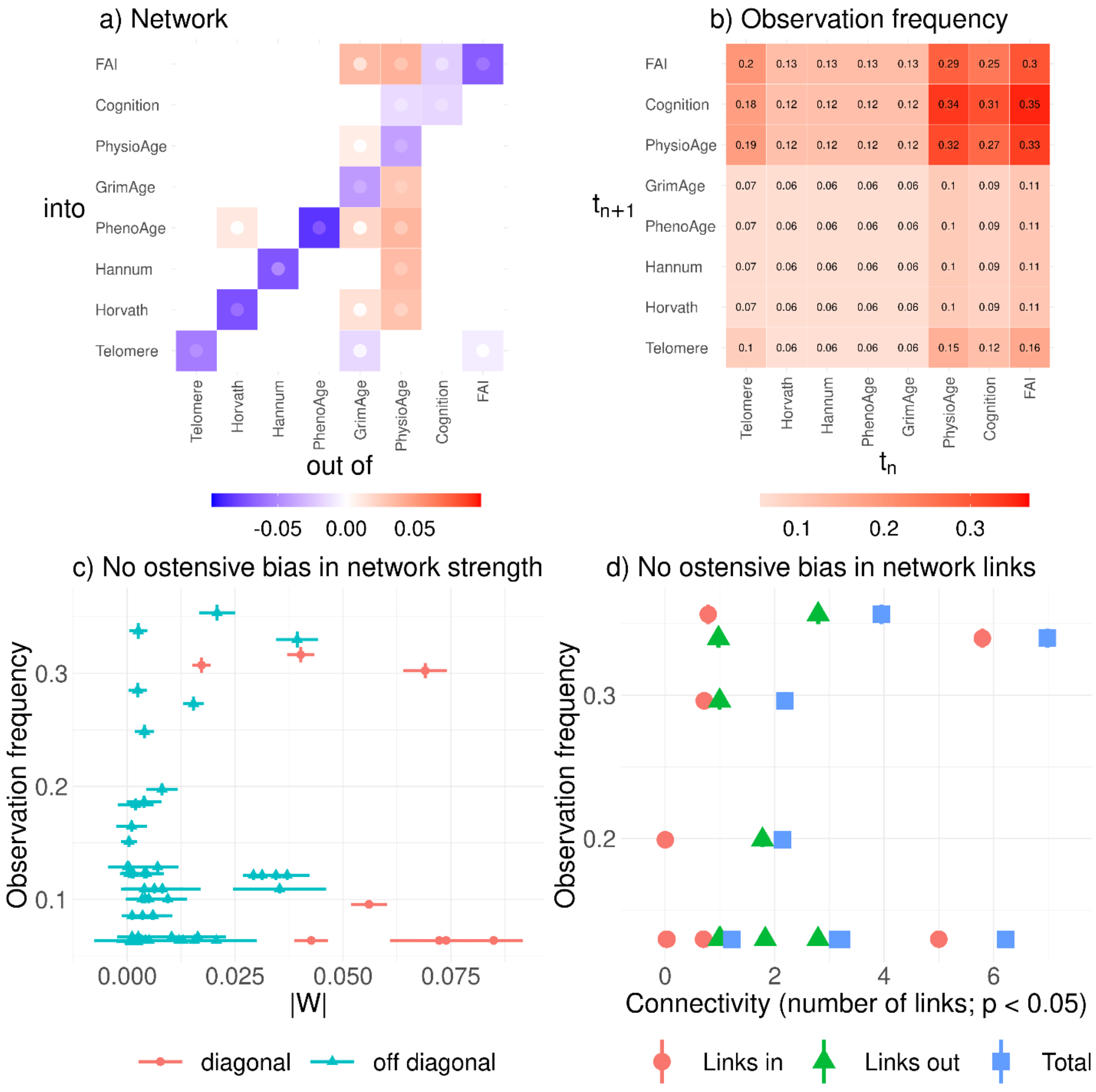

Figure S5: **Does the missingness bias the network estimation? a)** network estimate reproduced from Figure 1. Inner point is limit of 95% CI closest to 0: point is most visible for the least significant tiles. **b)** mutual observation frequency: each tile indicates how often x and y variables were both observed such that they could contribute to the log-likelihood. **c)** observation frequency versus magnitude $|W|$ shows no correlation in diagonal nor off diagonals suggesting that level of missingness does not affect the fit. **d)** significant links from **c** confirm that there is

no ostensive bias in the network due to missing values. Missing values were imputed as described in text.

We consider alternative missing data handling methods. We can use available case analysis using our previously derived iterative estimators[12]. These estimators are based on pairwise covariances and therefore we were able to estimate using all available pairs of values. The subsequent estimated network is reported in Figure S7. The network has a similar structure to the imputed case, in particular the same dominant central nodes: GrimAge and PhysioAge.

We also consider a different imputation algorithm. The use of multiple imputation permits us to estimate the uncertainty in imputed values using Rubin's rules[14]. By (correctly) estimating the error in the imputed values we can do no harm since any under-estimate in effect sizes introduced by the imputation should be compensated for by this increased error. That is, multiple imputation is "proper" so long as it is unbiased and has realistic error estimates[15]. We consider the effects of both missed measurements and dropout. The model we employ is a multilevel linear model where individuals are allowed their own slope, permitting individualized (linear) trajectories[15] (2l.pan[16]). When we imputed dropout we assumed those individuals' age trajectories continued forward until the end of the study, such that each individual had 9 timepoints. We used MICE (multiple imputation by chained equations) version 3.13.0 for R[16]. We included age and the FI in the imputation. We imputed each individual 20 times, since this number should be large enough to capture the underlying distribution of values while still being manageable. Each of the 20 imputations produced a separate dataset which we fit using the methodology outlined in the main text. Where specified we imputed dropout individuals, otherwise we exclude all values past dropout date. The parameters from each of these datasets, including the network estimates, were then pooled using Rubin's rules. This provides us with both an average estimate of the 'true' parameter values as well as an uncertainty estimate which includes stochastic effects via the bootstrap and uncertainty in the imputation via the multiple imputations.

We investigated the imputation quality of MICE both as individual trajectories (not shown) and at the population-level, Figure S6 (includes dropout imputation). In both cases the imputed values looked very similar to the observed values with similar mean and dispersion. The multiple imputations gave realistic error estimates for the range of possible values which a missing value could take. While this supports the conclusion that the MICE imputation better estimates the uncertainty in the parameter estimates, in does not necessarily indicate a better point estimate for the central (mean) parameter values. We know that the missing values are more likely to occur in the older individuals which suggests that the true values of the missing values should be older than the observed values. We don't see this effect in the MICE imputation (Figure S6), whereas we do with the expectation-maximization imputation (Figure S3): this can be seen by following

the trendlines which indicate a preference to impute data as older in Figure S3 whereas the trendlines coincide in MICE. Overall, however, the MICE imputation looked good.

We present the network estimates for four missing data handling methods in Figure S7 and the associated significance scores in Figure S8. We expect that the available case will under-estimate any effects and may be biased due to the non-random missingness (i.e. older, frailer individuals were more likely to be missing data). We further expect that the main result, which used expectation-maximization, will likely under-estimate errors and hence may over-estimate effect sizes. The MICE imputed networks should compensate for errors in the imputation process and hence should give realistic estimates for the true networks. Across the approaches we observe that GrimAge and PhysioAge are consistently the central nodes with the largest outgoing connections. This supports our primary interpretation of the network. We do see apparent differences between the diagonal strength in the main result and MICE but this is in the epigenetic ages and hence does not affect any of our conclusions. The available case notably showed a weaker role for PhysioAge and stronger role for Cognition in contrast to the other imputation strategies — which had a consensus central role for PhysioAge and minimal role of Cognition.

We also confirm that the natural variables yielded by MICE have similar properties to those in the main text. While we confirm this, it is worth noting that MICE-imputed values yielded natural variables which differ in a few interesting ways: Figure S9 vs Figure 3. Looking at the FI row we clearly see a strong propensity for health information to compress into the first few dimensions. While this effect is also seen in Figure 3, it is much cleaner in the MICE-imputed data which includes error estimates and an associated p-value cutoff at 0.05. The splitting of the first and second eigenvalues is also much stronger in the MICE data, which leads to bigger differences in the first and second natural variables.

Finally, we offer a note on our choice of imputation strategy. While MICE has the great advantage of permitting us to estimate the uncertainty in imputed values, we are concerned that it may have a stronger bias than our main choice (expectation-maximization). We had expected imputed individuals to look older since older individuals tended to have more missing data. We did see such an effect in the main approach (Figure S3) but did not in MICE (Figure S6). We also saw in Figure S20 that the age-stratified networks estimated by MICE were much more similar across age cohorts than were the main approach (Figure S19), which could indicate that the older individuals were made to look more like young individuals by MICE. We see two shortcomings of MICE: (1) it uses Fully Conditional Specification which is not formally self-consistent[15] as opposed to our main approach, and (2) due to the missingness bias, younger individuals had more data and may have therefore had more weight in the imputation model learned by MICE. While this latter point could also apply to our main result, the fact that we use an auto-regressive model should make imputed values less sensitive to parameter values. Regardless, both

imputation strategies yielded similar results and we can get some idea of the broader uncertainty in our results based on how the two compare.

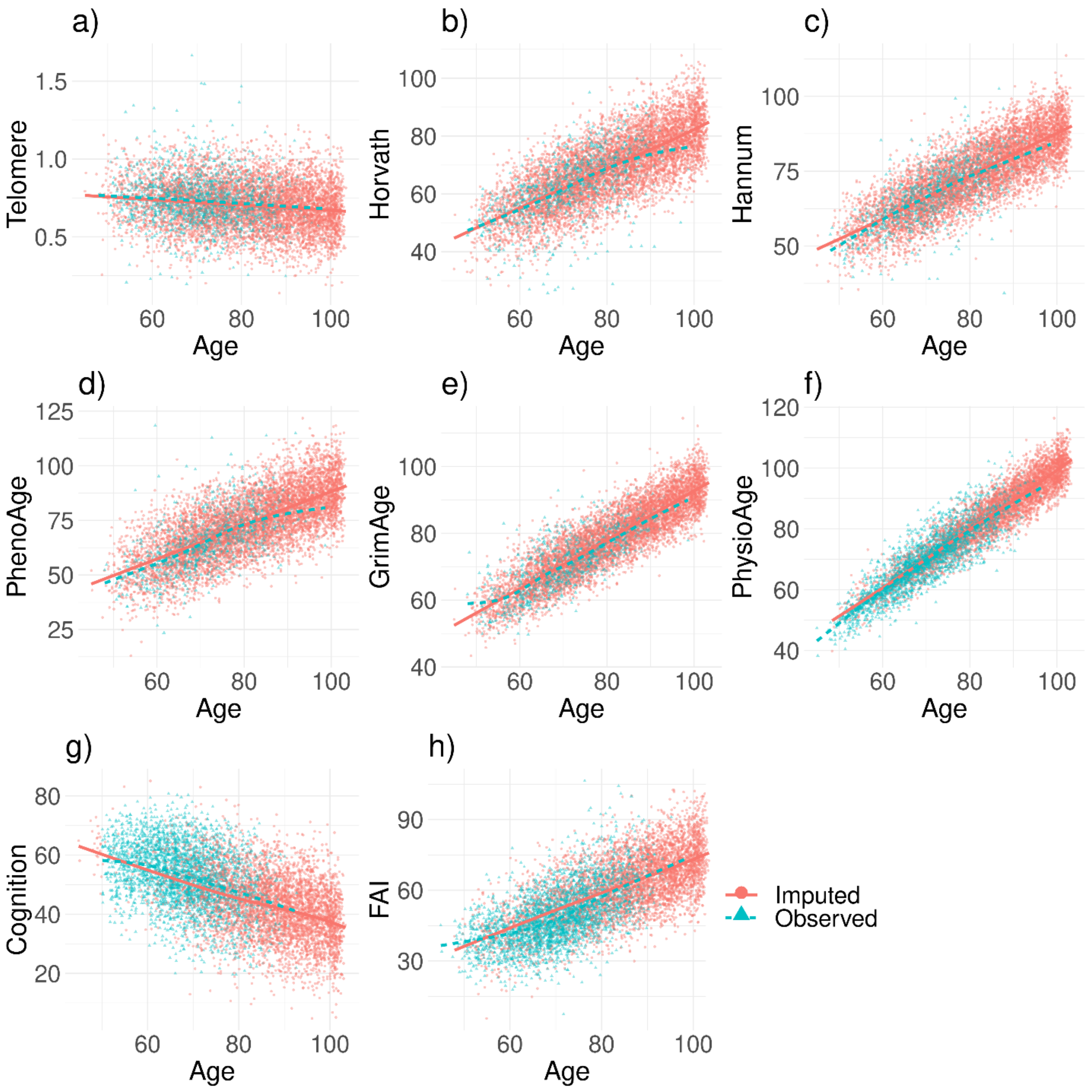

Figure S6: **MICE imputation quality (multilevel linear model with individual slope).** Population level data for all 8 BAs. Each individual was imputed 20 times and hence has 20 imputed values for each datum. As with Figure S3 we look for similar dispersion and trend with the imputed (red dots) and observed (blue triangles) values. In contrast to Figure S3, we have also imputed values after dropout which we then optionally removed (hence the values extend to older ages). We see nearly identical trends between the imputed and observed values: there is a best fit line for each, on each plot, but they typically overlap. We also see realistic dispersion.

These are all good signs. Note, however, that we know that the missing values are biased towards older individuals so we would expect that the best fit line for the imputed should be a bit higher than the observed – which we see in Figure S3 but not here. It is therefore unclear which of the two offers the better imputation. The advantage of MICE is that it quantifies the uncertainty in the imputation using Rubin's rules.

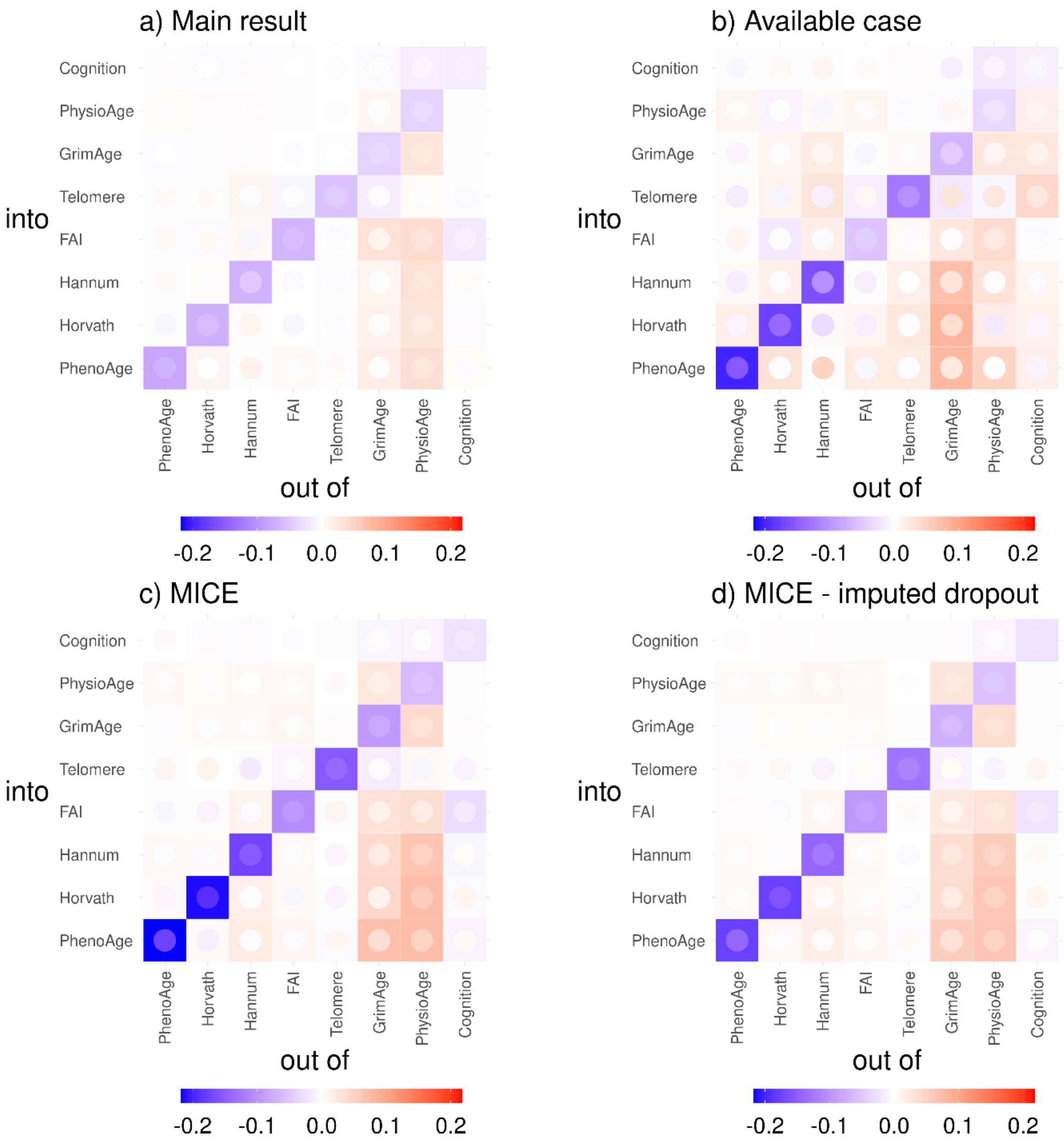

Figure S7: **Effect of missing data handling on network estimate.** We compare 4 missing data handling strategies (**a-d**). In all cases we see strong outgoing links from GrimAge and PhysioAge, supporting our main result which implicates them as the central nodes. We expect

that the MICE imputation **(c)** will give the best confidence estimates since it utilizes multiple imputation and Rubin's rules to estimate the uncertainty in imputation. By including dropped individuals, **d)**, we can also surmise the effect that censorship has on the network — which is clearly negligible (**c** and **d** look almost identical). Inner point is limit of 95% CI closest to 0: point is most visible for the least significant tiles; if point is opposite colour to tile then element is not significant. See Figure S8 for link significance (z-scores).

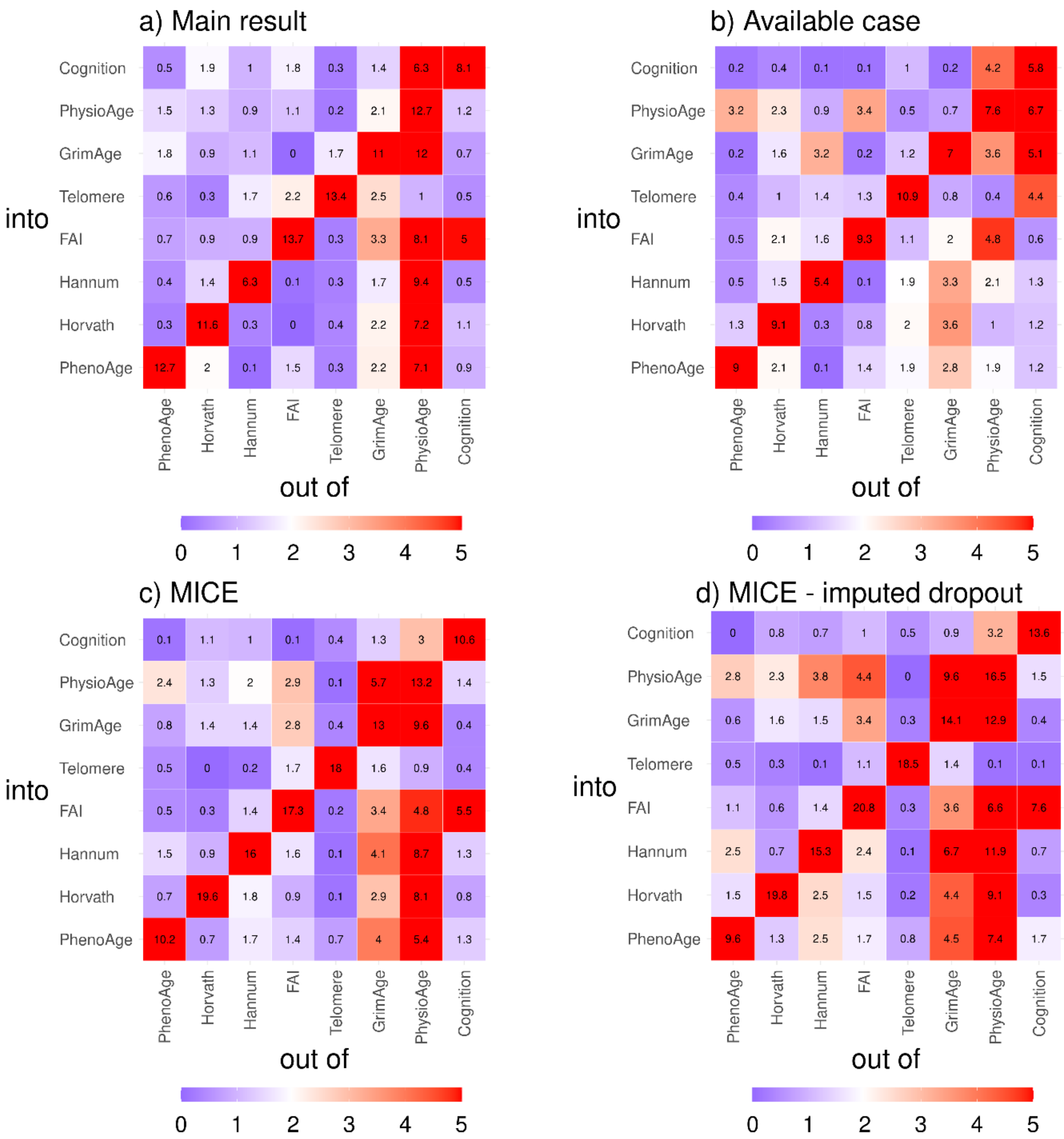

Figure S8: **significant network links by missing data handling method (z-scores)**. Blue are non-significant, red and white are significant at $p < 0.05$ ($z > 1.96$). Standard errors were

estimated by bootstrap (100 repeats). Observe the very high significance of the links outgoing from PhysioAge, supporting its central role in the network.

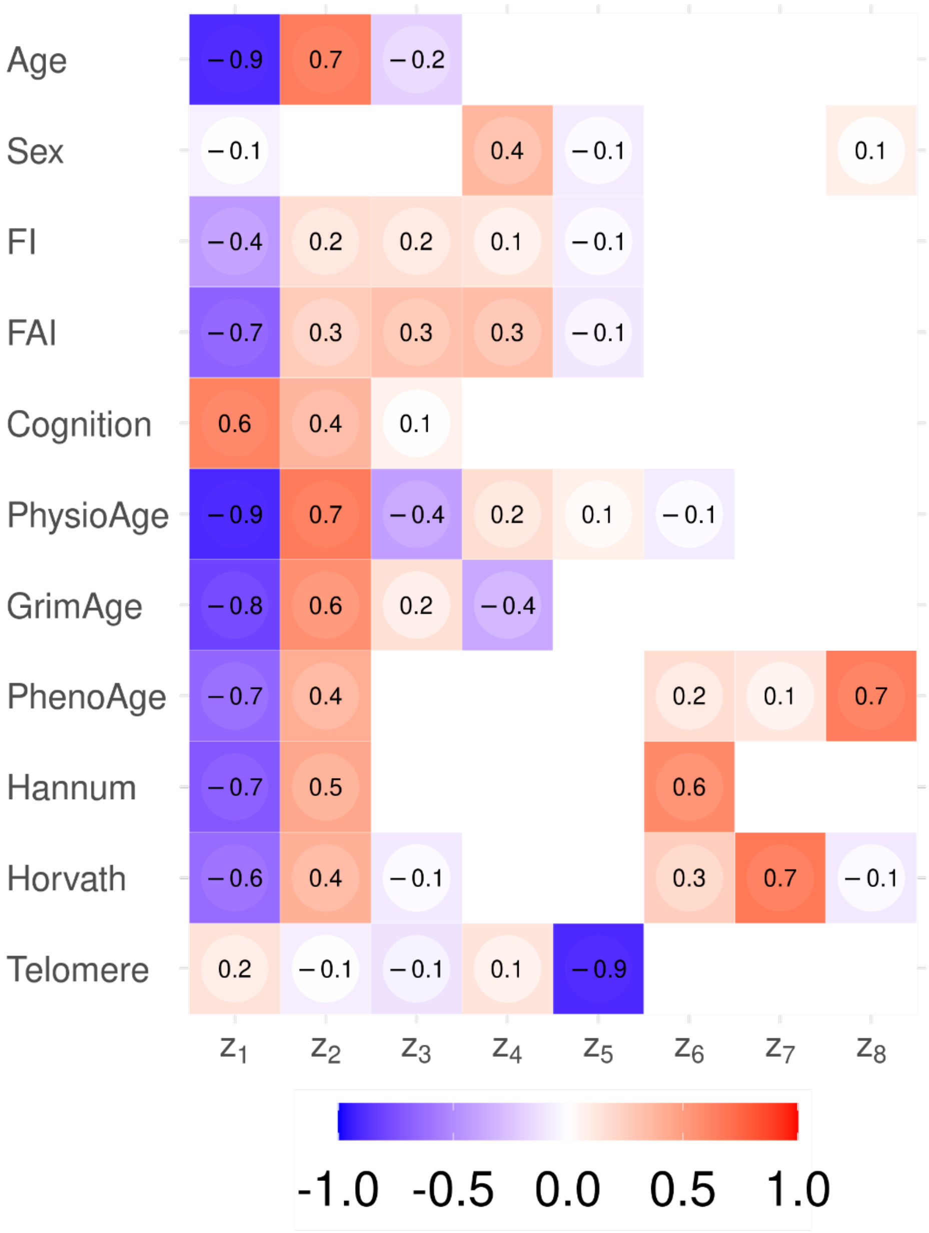

Figure S9: **Natural variable correlates — MICE**. Spearman correlations between the natural estimates and the original variables for the MICE-imputed data. Errors were estimated by 100 repeat bootstrap. Non-significant tiles by z-scores are whited-out ($p > 0.05$). Inner point is limit of 95% CI closest to 0: point is most visible for the least significant tiles.

Estimation

We previously reported an iterative estimator as well as a diagonal-space weighted linear regression estimator for the Stochastic Finite-difference (SF) model[12]. Here we show that the linear regression estimator applies more generally, including for all invertible $\boldsymbol{W}$. The linear estimator is always valid for $\boldsymbol{W}$ but can only estimate $\boldsymbol{\Lambda}$ and $\vec{\mu}_0$ if $\boldsymbol{W}$ is invertible ($\boldsymbol{W}$ can be stable or unstable but cannot have any eigenvalues equal to 0). Usually this is not a problem since real data is unlikely to have an eigenvalue of exactly 0, but we can nevertheless circumvent the problem entirely by pre-processing using principal component analysis (PCA), since this will allow us to drop reduced-rank terms (i.e. eigenvalues close to 0). The final parameters can then be mapped back into observed space using the PCA transformation.

Recall that our SF model is written as

$$\begin{aligned} \vec{b}_{n+1} &= \vec{b}_n + \boldsymbol{W}\Delta t_{n+1}\left(\vec{b}_n - \vec{\mu}_n\right) + \vec{\epsilon}_{n+1} \\ \vec{\epsilon}_{n+1} &\sim N(0, \boldsymbol{\Sigma}|\Delta t_{n+1}|) \\ \vec{\mu}_n &\equiv \vec{\mu}_0 + \boldsymbol{\Lambda}\vec{x}_n \end{aligned} \tag{S8}$$

which can be rewritten

$$b_{jn+1} - b_{jn} = \sum_k W_{jk}\,\Delta t_{n+1} b_{jn} - \sum_{k,l} W_{jk}\,\Delta t_{n+1}\Lambda'_{jl} x_{ln} + \epsilon_{jn+1} \tag{S9}$$

for each BA, where $\boldsymbol{\Lambda}'$ includes $\vec{\mu}_0$ by inventing a new $x_n = 1$. This can be rewritten in a more revealing way as

$$\Delta b_{jn+1} = \boldsymbol{W}_{j\cdot}\vec{\alpha}_n + \mathbf{A}_{\mathrm{j}\cdot}\vec{\beta}_n + \epsilon_{jn+1} \tag{S10}$$

where $\alpha_{jn} \equiv \Delta t_{n+1} b_{jn}$, $\beta_{jn} \equiv \Delta t_{n+1} x_{jn}$, $\mathbf{A} \equiv -\mathbf{W}\boldsymbol{\Lambda}$ and $\Delta b_{jn+1} \equiv b_{jn+1} - b_{jn}$ ($\boldsymbol{W}_{j\cdot}$ is the jth row of $\boldsymbol{W}$). Eq. (S10) is a (weighted) linear regression equation (note: because of the noise you must weight each entry by $1/|\Delta t_{in+1}|$ as well[12]). These regression equations estimate $\boldsymbol{W}$ and the product $\mathbf{A} = -\boldsymbol{W}\boldsymbol{\Lambda}$, hence we must be able to invert $\boldsymbol{W}$ to estimate $\boldsymbol{\Lambda}$ using linear regression ($\det(\boldsymbol{W}) \neq 0$). We used Eq. (S9) to estimate our model parameters $\boldsymbol{W}$ and $\boldsymbol{\Lambda}$ ($\vec{\mu}_0$ was estimated via $\boldsymbol{\Lambda}$ by inventing a constant $x_n \equiv 1$). The noise, $\boldsymbol{Q} = \boldsymbol{\Sigma}^{-1}$ was estimated using the model residual[12].

Note that although the error terms are coupled across variables, this does not affect estimation.

Proof

Consider the (equivalent) simplified form of our problem,

$$\vec{y} = \boldsymbol{W}\vec{v} + \vec{\epsilon} \tag{S11}$$

where $\vec{\epsilon} \sim N(0, \boldsymbol{\Sigma})$. Because $\boldsymbol{\Sigma}$ is a covariance matrix it must be positive definite and hence there exists a transformation $\boldsymbol{P}$ which diagonalizes $\boldsymbol{P^T\Sigma P} = \boldsymbol{\Gamma}$ with $\mathbf{P^T} = \mathbf{P^{-1}}$ for diagonal $\boldsymbol{\Gamma}$. Hence $\boldsymbol{P^T}\vec{\epsilon} \sim N(0, \boldsymbol{\Gamma})$. This means we can transform Eq. (S11) such that the equations decouple and can safely be estimated independently,

$$z_j = \widetilde{\boldsymbol{W}}_{j\cdot}\vec{u} + \gamma_j \tag{S12}$$

Where $\widetilde{\boldsymbol{W}} \equiv \boldsymbol{P^T W P}$, $\vec{\gamma} \equiv \boldsymbol{P^T}\vec{\epsilon}$, $\vec{z} \equiv \boldsymbol{P^T}\vec{y}$ and $\vec{u} \equiv \boldsymbol{P^T}\vec{v}$. The least squares estimator is[17]

$$\widetilde{\boldsymbol{W}}_{j\cdot} = \left(\sum_i \vec{u}_i\vec{u}_i^T\right)^{-1} \sum_i \vec{u}_i z_{ij}$$

$$\widetilde{\boldsymbol{W}} = \left(\sum_i \boldsymbol{P^T}\vec{v}_i{\vec{v}_i}^T\boldsymbol{P}\right)^{-1} \sum_i \boldsymbol{P^T}\vec{v}_i\vec{y}_i^{\boldsymbol{T}}\boldsymbol{P} \tag{S13}$$

$$\widetilde{\boldsymbol{W}} = \boldsymbol{P^T}\left(\sum_i \vec{v}_i{\vec{v}_i}^T\right)^{-1} \sum_i \vec{v}_i\vec{y}_i^T\,\boldsymbol{P} \tag{S14}$$

$$\boldsymbol{W} = \left(\sum_i \vec{v}_i{\vec{v}_i}^T\right)^{-1} \sum_i \vec{v}_i\vec{y}_i^T, \tag{S15}$$

which is exactly the ordinary least squares estimator of Eq. (S11).

QED

Additional Results

Model Selection

Here we provide additional results that were not included in the main text for want of space. First we demonstrate that our model works. In Figure S10 we compare 3 variants of our model to simply carrying forward the previous value to predict the future value (equivalent to $\boldsymbol{W} = \mathbf{0}$). The fully flexible model is FullW, whereas the other models are simplified variants to reduce overfitting, specifically: assume that $\boldsymbol{W}$ is diagonal (DiagW) or assume that $\boldsymbol{W}$ is diagonal in PC-space (SymW, where PCA forces $\boldsymbol{W}$ to be symmetric because PCA is an orthogonal transformation). In both FullW and SymW we first performed a PCA transformation to avoid collinearity issues and potential fit problems due to reduced-rank $\boldsymbol{W}$ (the transformation was learned using timepoint 1). We use 632-corrected root mean squared error (RMSE) and mean absolute error (MAE) to quantify error (lower is better); e.g.[17] $\mathrm{RMSE}_{632} \equiv 0.632 \cdot \mathrm{RMSE}_{\mathrm{test}} + 0.368 \cdot \mathrm{RMSE}_{\mathrm{train}}$ (632-correction reduces bias[12]). We observe that all of our models out-perform forward carry (in which all values are carried forward unchanged to the next timepoint). Additionally, FullW and SymW out-performed DiagW, indicating that an interaction network is supported by the data.

Whereas the FullW model includes directed links, SymW is a parsimonious model that permits only undirected links. While FullW appeared to have a lower MAE, the RMSE was the same as SymW — within error. To break the tie, we consider a simplified accuracy measure: which model best predicts worsening. In Figure S11 we present the AUC[18] for correctly predicting worsening. We observe that a causally unambiguous model (FullW) out-performs the symmetrical $\mathbf{W}$ (SymW) at $p = 0.1$ (Delong test[18]). Hence, for both the MAE and the AUC we find FullW out-performs SymW at 68% confidence (non-overlapping error bars). If the two metrics (MAE and AUC) are independent we have $p = 0.1 \cdot (1 - 0.68) = 0.04$ which is significant at 95% confidence. Given the evidence, we selected FullW as the best model.

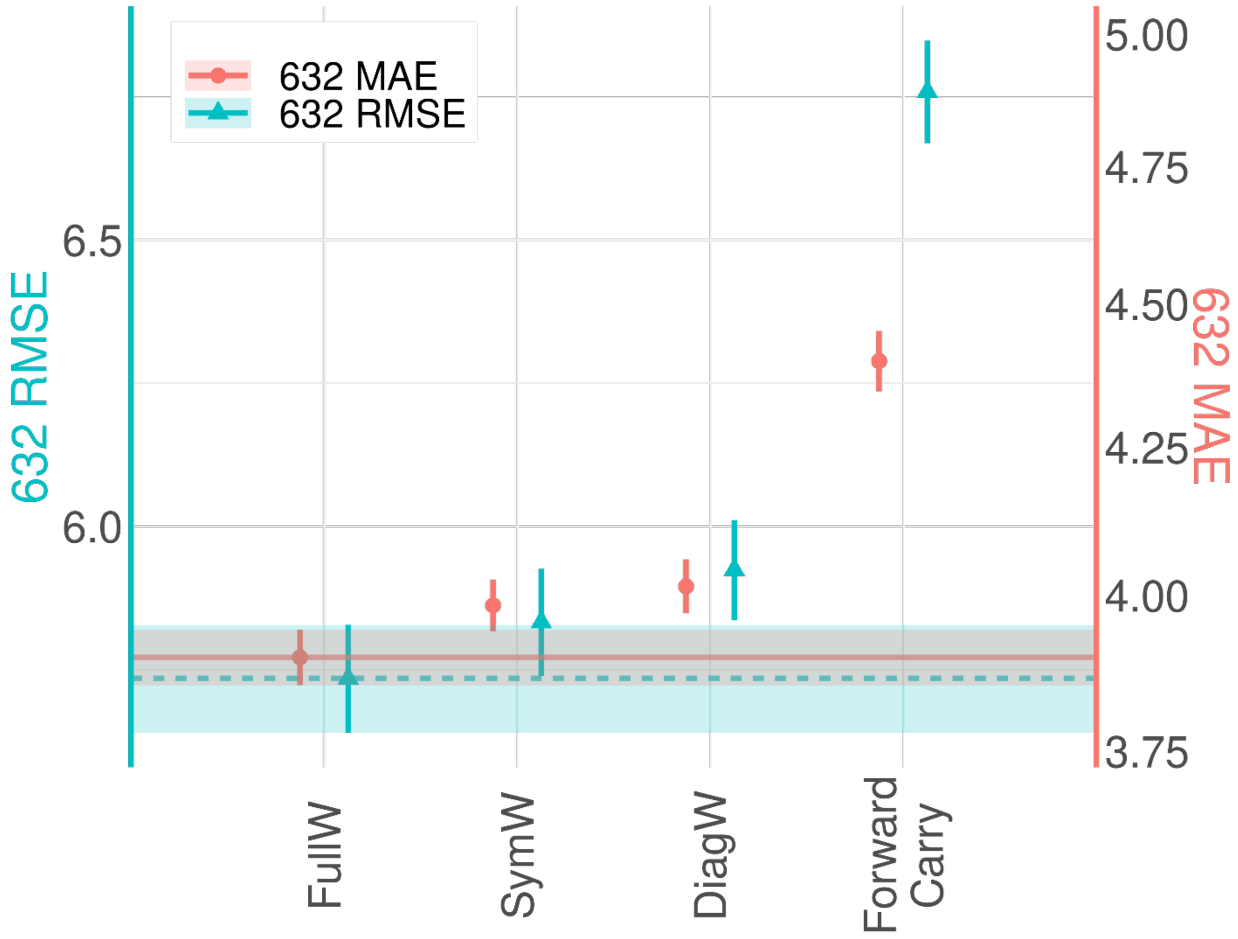

Figure S10: **Model error, lower is better.** Our model easily out-performs forward carry. The diagonal $\boldsymbol{W}$ (DiagW) performs worse than the full $\boldsymbol{W}$ (FullW) for both MAE and RMSE (bands). The symmetric $\boldsymbol{W}$ (SymW), on the other hand, performs worse in MAE but not RMSE. Scale is years of age. Error bars are standard error.

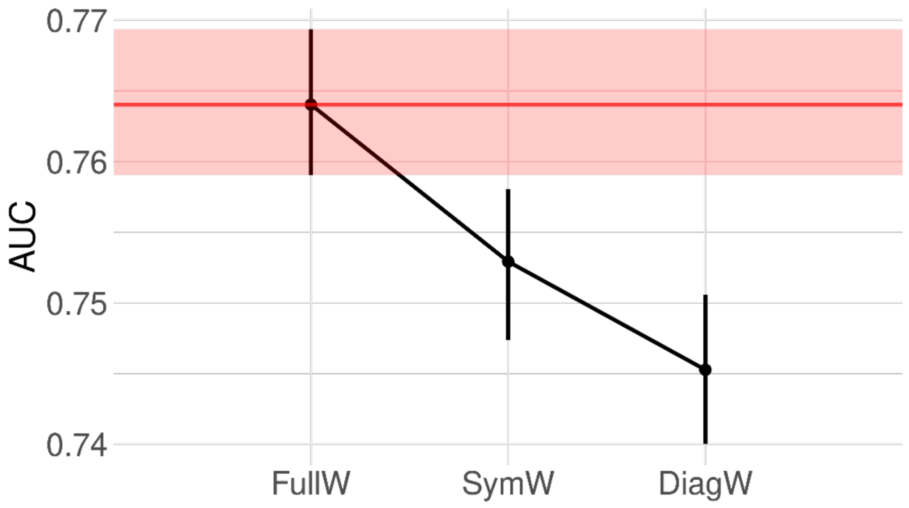

Figure S11: **Worsening detection model performance**, higher is better (0.5: guess, 1: perfect). The symmetric $\boldsymbol{W}$ model (SymW) performed worse than the model which includes a fully flexible $\boldsymbol{W}$ (FullW), $p = 0.1$ (Delong test[18]). The diagonal model (DiagW) performed worse than FullW, $p = 0.01$ (Delong test[18]). Forward carry did not perform better than a guess (AUC: 0.501 ± 0.006, not shown). Error bars are standard errors using N=2000 resample bootstrapping[18]. Individuals worsened in 60.0% of measurements (they improved in 40.0% of measurements).

Model Parameters

The full model estimates an unconstrained matrix of network weights, **W**, together with equilibrium state $\vec{\mu}_n$ which depends on sex, and a positive-definite stochastic noise term, **Σ**. Whereas **W** is presented in the main text, $\vec{\mu}_n$ (Table S1) and $\boldsymbol{\Sigma} = \mathbf{Q}^{-1}$ (Figure S12) are presented below. The exact model parameters are provided in CSV file format in the GitHub repository, these can be used to simulate interventions https://github.com/GlenPr/stochastic_finite-difference_model.

The equilibrium state is determined by $\vec{\mu}_n$, although the system converges if and only if $\lambda_k < 0$ (and at a rate of $-\lambda_k$). In Table S1 we present the coefficients of $\vec{\mu}_n$, including the intercept, $\vec{\mu}_0$ and the sex-dependence (Λ_{sex}). The sex-dependence is weak, reflecting that the model isn't sensitive to starting positions for each individual at their baseline (the sex dependence appears to be small and due to starting position[3]). Observe that *in all cases* the μ_{j0} is much different from the population age range (45-88) and always in the risk direction. This ensures that all individuals drift towards worsening BAs as they move towards the equilibrium position. The equilibrium positions are far enough away that they will never be reached since the individuals

will die long before that happens. The errors are large, ostensibly because it is important that μ_{j0} is far away but less important exactly how far away. Figure S14 illustrates.

Table S1: equilibrium positions, μ_0 and $\boldsymbol{\Lambda}$ (units of years).

Biological age	Risk Direction[1]	μ_0	$\Lambda_{sex}^{(2)}$
Telomere	Down	35 ± 24	4 ± 3
Horvath	Up	127 ± 38	-5 ± 4
Hannum	Up	130 ± 35	-5 ± 4
PhenoAge	Up	134 ± 41	-4 ± 4
GrimAge	Up	136 ± 37	-6 ± 4
PhysioAge	Up	149 ± 43	-4 ± 5
Cognition	Down	-122 ± 107	10 ± 12
FAI	Up	190 ± 82	-4 ± 9

(1) Direction of change with increasing chronological age.

(2) Male: 0, female: 1 (coefficient modifies only females).

Our model captures stochastic effects via the noise term, $\boldsymbol{\Sigma}$. In Figure S12 we present the normalized $\boldsymbol{\Sigma}$, which reflects correlations between the biological ages in their response to stochasticity: stressors, individual variabililty, and non-linearities. We observe three self-evident blocks, representing three scales: telomere noise does not correlate with the other biomarkers, epigenetic ages all mutually correlate strongly along with PhysioAge, and finally Cognition and FAI correlate mutually. These blocks suggest that stochastic effects are not shared across scales. The exception appears to be PhysioAge and GrimAge which both couple to Cognition and FAI. Between $\boldsymbol{W}$, which is related to the resilience via recovery rate, and $\boldsymbol{\Sigma}$, which is related to robustness via stressor effects, we consistently observed central roles for PhysioAge and GrimAge: for connecting, and potentially driving, the changes observed in the other BAs.

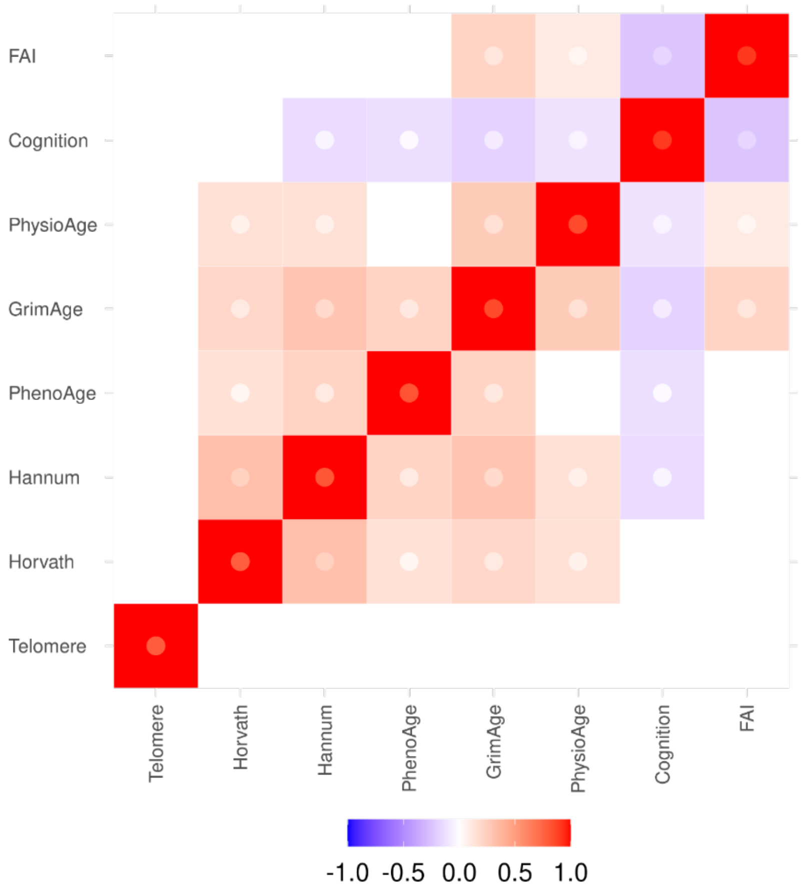

Figure S12: **Normalized noise matrix, $\boldsymbol{\Sigma}$**. Normalized by diagonal strength. Note the overlapping, block-diagonal structure. The blocks suggest common domains while the overlapping ages may represent feedbacks between domains, i.e. GrimAge, PhysioAge and, to an extent, Cognition. Inner point is limit of 95% CI closest to 0: point is most visible for the least significant tiles. Non-significant tiles are whited-out ($p > 0.05$).

Natural Variables

Eigen-decomposition of the interaction network, $\boldsymbol{W}$, yields a transformation matrix of eigenvectors ($\boldsymbol{P}$ in Eq. (S7)). The eigenvector represents canonical coordinates which decouple the mean-interactions, greatly simplifying the dynamics (they now satisfy Eq. (3)). The eigenvector transformation matrix, $\boldsymbol{P}$, can be used to generate scores for each of the i individuals using their data vector, $\vec{b}_{in}$, similar to the way in which PCA generates PC scores. This generates natural aging variables, $\vec{z}_{in} \equiv \boldsymbol{P}^{-1}\vec{b}_{in}$, which are aggregated BAs. The age-dependence of the natural variables are plotted in Figure S13; for comparison, the BAs are plotted in Figure S14.

It is noteworthy that the strongest correlations with age are present in the lowest z_j, suggesting that the aging phenomenon is concentrated into the slowest recovery dimensions (recall z_j has the jth slowest recovery). Also observe the positions of the equilibrium positions, μ_j. In the BA-picture (Figure S14), μ_j was always far away in the risk direction, ensuring that each BA drifted continuously for each individual during their lifespan. In the z-picture, equilibrium is quickly reached for all of the fast recovering z_j, with the slower z_3 equilibrating around age 80 and z_1 and z_2 never reaching equilibrium. We previously observed this same phenomenon for health biomarkers[12]. This has two effects: (1) the mean continues to drift indefinitely, causing the slow z_1 and z_2 to become increasingly dominant in the mean as individuals age, and (2) the variance will also typically continue to increase. Together this means that at advanced ages z_1 and z_2 can dominant both the mean and variance, meaning that they dominate what we observe in the BAs since they are connected via the transformation $\boldsymbol{P}\vec{z}_{in} \equiv \vec{b}_{in}$. Hence the z_j are natural *aging* variables since they become increasingly simple, i.e. low-dimensional, as individuals age.

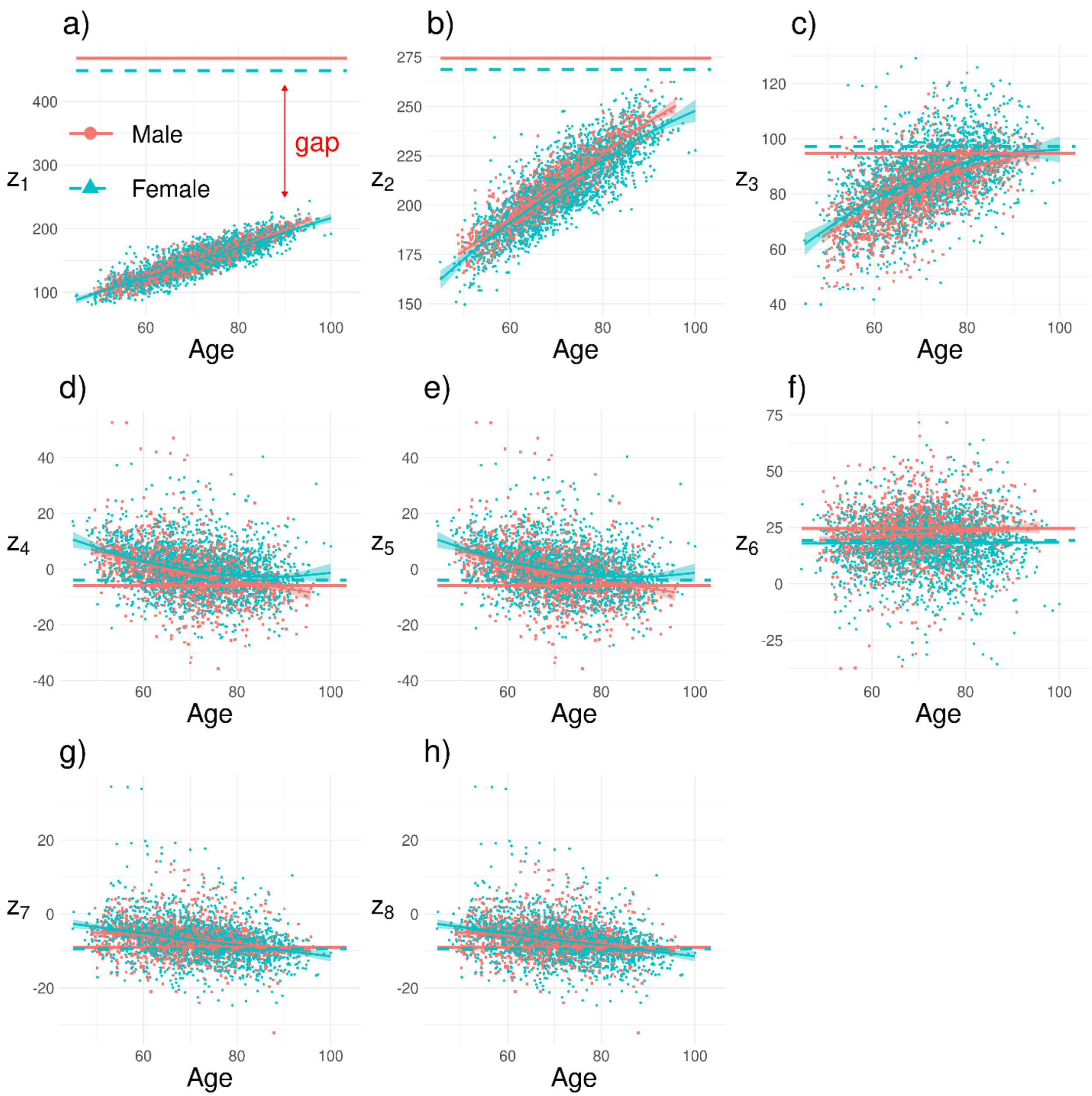

Figure S13: **natural variable age-dependencies, together with equilibrium position (μ_j).** In contrast to the BAs (Figure S14), most of the natural variables equilibrate at around age 80 where they cross the horizontal equilibrium line, μ_j. The exceptions are z_1 and z_2 which both have gaps between the observed values and μ (e.g. as indicated for z_1), indicating that they never equilibrate, instead drifting up for the entire human lifespan. This constant drift explains why z_1 and z_2 have the strongest age-dependence. We see little sex effects, primarily concentrated into z_3. Real components only. Note: z_4/z_5 and z_7/z_8 are conjugate pairs which differ only in their imaginary component. Lines are cubic splines from the MGCV package with default parameters[13].

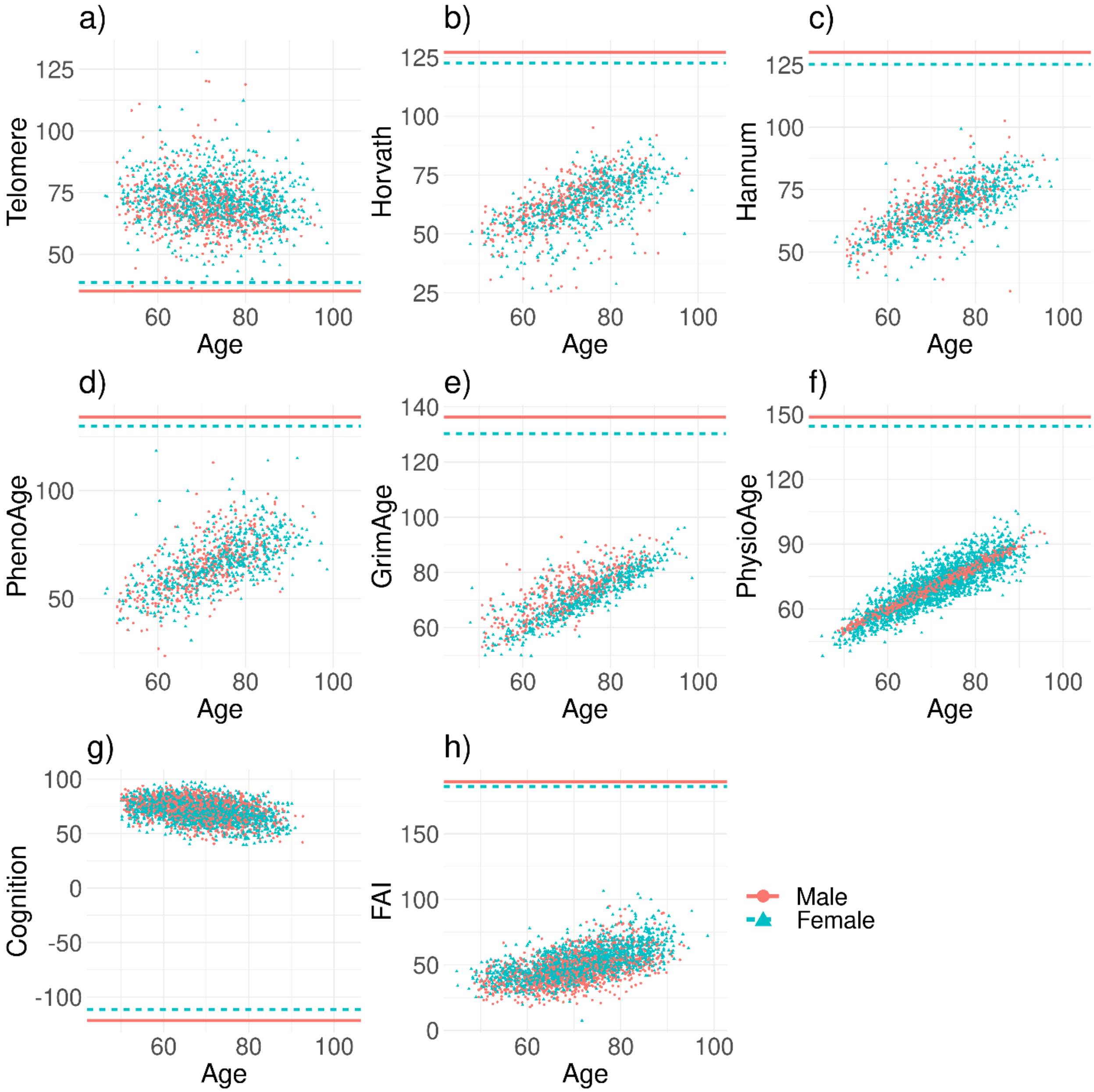

Figure S14: **biological age (BA) age-dependencies, together with equilibrium position (μ_j).** Each BA is far from equilibrium (horizontal lines), and will never reach it during a normal human lifespan. Compare to the natural variables, which compress this non-equilibrium behaviour into the first two variables (Figure S13). Sex effects are relatively small (red points/solid lines vs blue triangles/dashed lines).

Simulated Interventions

Once we have estimated the model parameters we can simulate new data. We introduce interventions into the simulated data as instantaneous rejuvenations, emulating the rapid switching which occurs in some anti-aging interventions, e.g. dietary restriction of flies[19]. (Interventions may not look instantaneous in the figures because of the finite step size (1 year) in the simulation.) In the main text we focused on a simple intervention which rejuvenates PhysioAge by 10 years, applied at age 70. Here we considered modifications of the intervention.

First we considered instead worsening PhysioAge by 10 years. This could represent the effects of disease, for example, severe COVID is associated with persistent cognitive decline of strength comparable to aging 10 years[20]. The simulated effect is identical to that of rejuvenation, with the sign flipped, Figure S15b. We have also included a visualization of the effect of the intervention on the health trajectory of the population in Figure S15a.

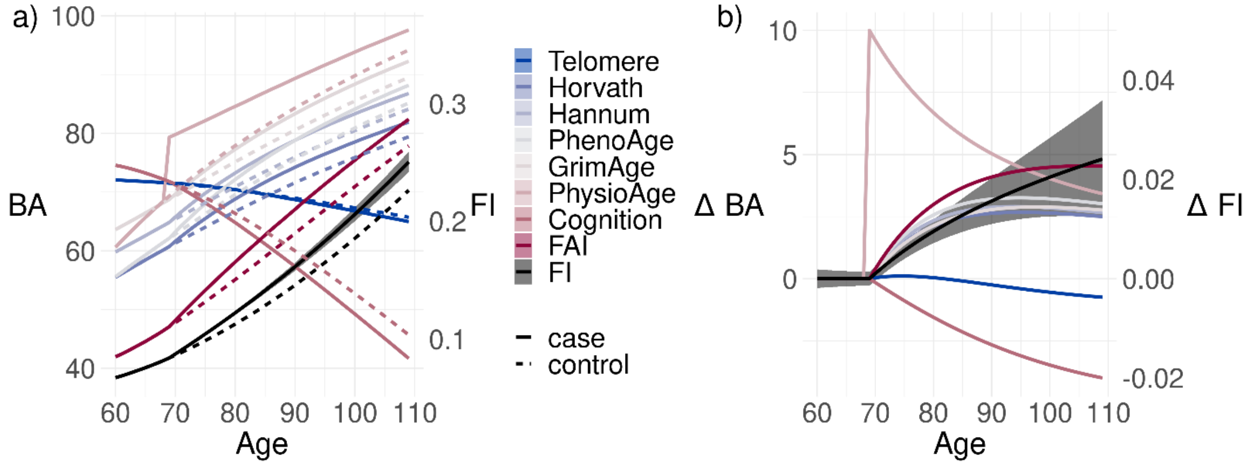

Figure S15: **Simulated harmful intervention on PhysioAge.** We simulated a hypothetical intervention at age 70 which instantly ages PhysioAge by 10 years. The effects are identical to the rejuvenation (Figure 4) but with the sign flipped. Step size: 1 year. Band is standard error (often smaller than line width). The FI has its own y-scale as indicated on the right-hand side.

Both the rejuvenation and worsening of PhysioAge show delayed and transient effects in the other BAs due to the information of the intervention propagating from PhysioAge throughout the network. The natural variables, z_k, have very different dynamics. This is because they are eigenvectors ("normal modes") which do not share information (in the mean). While multiple z_k can receive correlated information through the noise, that information averages to 0. As a result, intervening on any single z_j leaves the other z_k permanently unaffected, Figure S16b. All BAs driven by z_1 via $\vec{b} = \boldsymbol{P}\vec{z}$ are commensurately affected by the intervention, Figure S16a. Because z_1 is well-connected, it drives *all* of the BAs when intervened upon. This means that the

interventions on z_1 appear optimal, but can look complicated in the BAs — only when monitored in the z-picture do they appear simple.

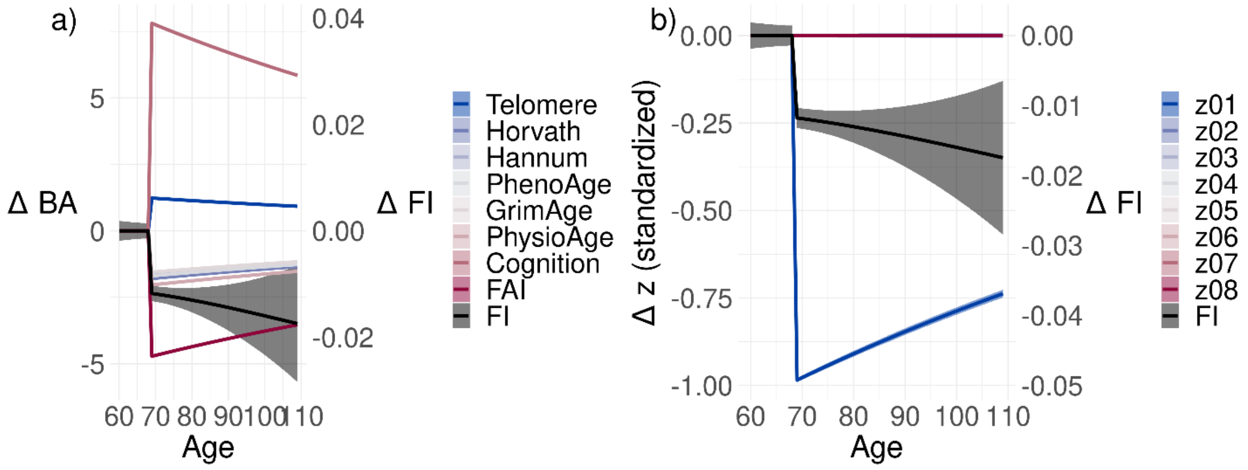

Figure S16**: Simulated intervention on the least stable natural variable, z_1**. We simulated a hypothetical intervention at age 70 which instantly rejuvenated z_1 by 10 years. The z_j are dynamically independent hence the intervention never affects the $z_{j\neq1}$, greatly simplifying its effects. The intervention is equivalent to intervening on all of the BAs with weighs equal to $P_{\cdot 1}$ (because $\vec{b} = \boldsymbol{P}\vec{z}$). The FI continues to improve after intervention, since it is unstable and the initial change compounds with age. Step size: 1 year. Band is standard error (often smaller than line width). The FI has its own y-scale as indicated on the right-hand side.

For those interested in simulating their own interventions, the GitHub page includes parameters in CSV files and the code needed for simulating interventions. For simulation starting values, we sampled real values[3], but we alternatively provide multivariate normal starting statistics in the repository.

Sensitivity Analysis

We tested the robustness of our key results, i.e. the network topology, by fitting with modified data. We considered splitting the males and females, and also we considered adding the transformed FI as a BA and including CA (chronological age).

Sex-specific Networks

We considered the confounding effect of sex by separately fitting to males and females. Our key results were unchanged: the networks were similar to the pooled fit and to each other (Figure S17) and showed nearly identical eigenvalues (Figure S18). In particular, both males and females showed 2 low-stability eigenvalues. Males and females also showed high-connectivity of PhysioAge, and to a lesser extent GrimAge. The only noteworthy difference was the strength of the outgoing links from PhysioAge, which was higher in males. When building PhysioAge, males and females were separately calculated using different covariates, following feature selection[3]. Hence these differences may simply reflect that a different definition for PhysioAge was used for males versus females.

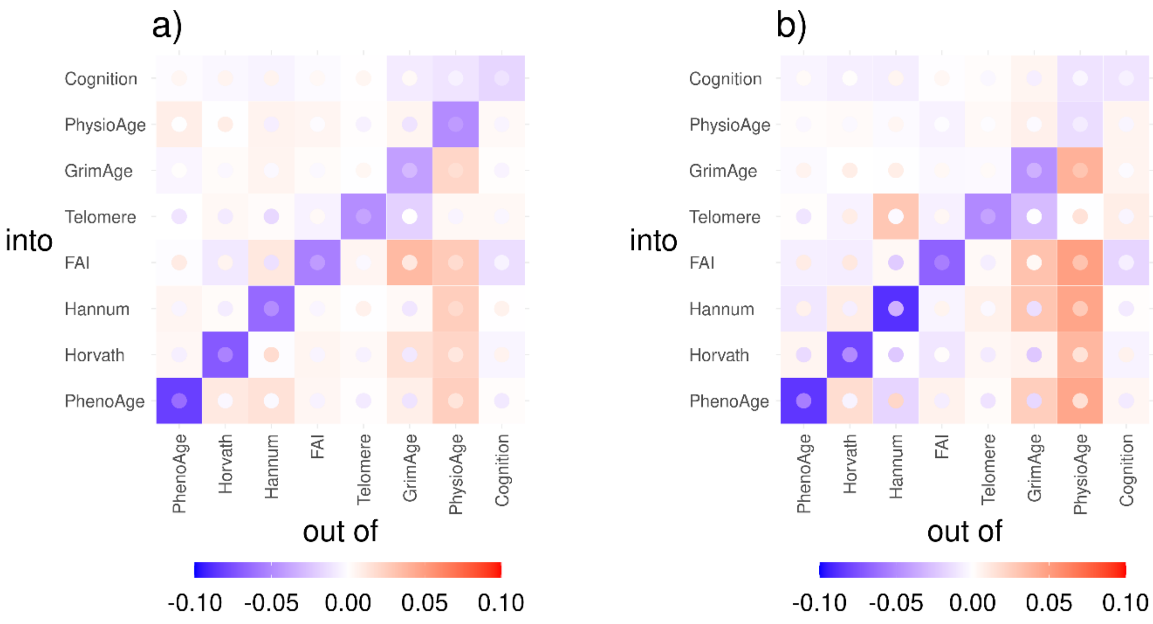

Figure S17: **Female vs male interaction networks**. While females **(a)** and males **(b)** share common network structures, there were notable differences. In particular, while PhysioAge was the main driver in both sexes, males had notably stronger connections from PhysioAge to the BAs. This may reflect that different biomarkers were used to construct the male and female PhysioAges. Inner point is limit of 95% CI closest to 0: point is most visible for the least significant tiles; if point is opposite colour to tile then element is not significant.

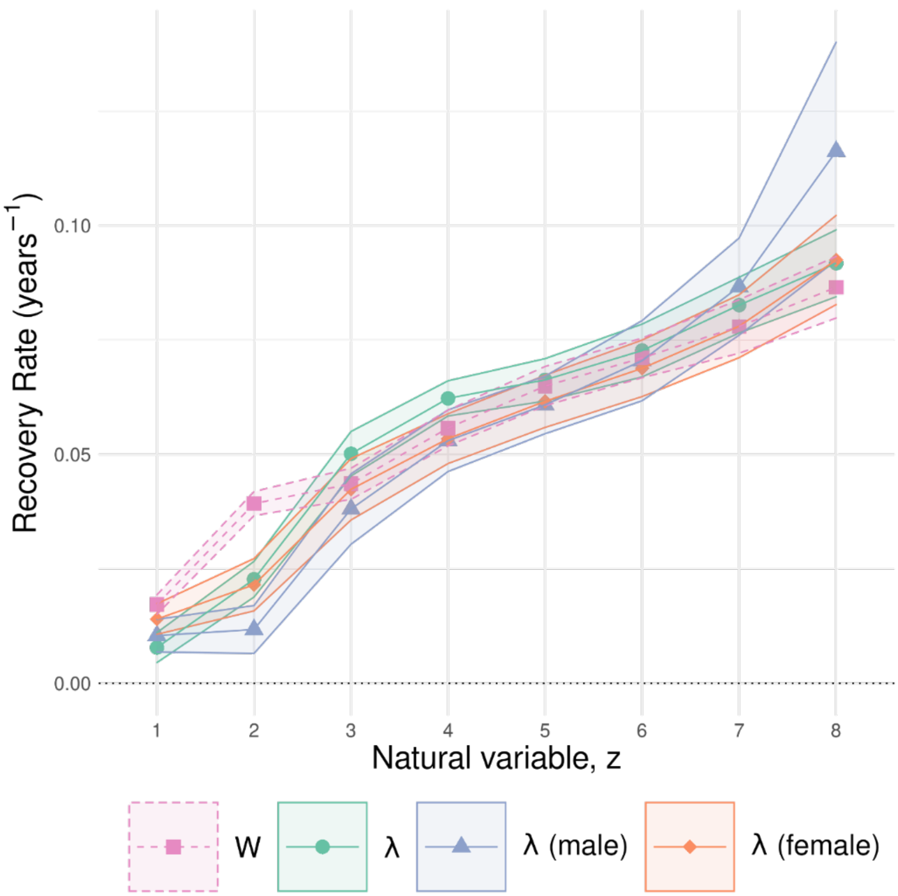

Figure S18: **Network stability (resilience) including sex.** We observe 2 weakly stable eigenvalues which are notably less stable than the least stable network diagonal elements (W_{ii}). No strong sex effects are apparent. Bands are standard error.

Age-specific Networks

Do the $\boldsymbol{W}$ matrix connections and/or stability change with age? Others have observed age-dependent changes to short timescale resilience[21], or have predicted that long timescale stability will decrease with age[22]. Our estimator is linear and hence does not require a great deal of data to fit with[12]. This permits us to check for age-dependent changes by splitting up the population into age cohorts which are fit separately.

An important consideration is that the missingness was age-dependent, particularly in the epigenetic ages and hence the older ages will be the hardest to estimate and therefore most prone to bias from the imputation. Including dropout, the youngest quartile had 64% missingness including 80% missingness of each epigenetic age versus the oldest quartile which had 87% missingness including 93% missingness of each epigenetic age.

We split up the population into four cohorts based on the baseline age quartiles then fit our model using the usual methodology outline in the main text. In Figure S19 we present the fitted networks using our expectation-maximization imputation strategy from the main text. The MICE imputed data yielded cleaner results, although we are concerned it may have removed the age dependent structure in the data (Figure S20).

The stability is plotted for both the main imputation strategy and MICE in Figure S21. For the main approach we again see an indication that stability may be dropping with age. This change isn't very large relative to errors, however, and is not present in the MICE data.

We see no clear age dependence. We suspect that there is simply too much missing data and not enough individuals/timepoints to confirm or reject any age-dependent changes.

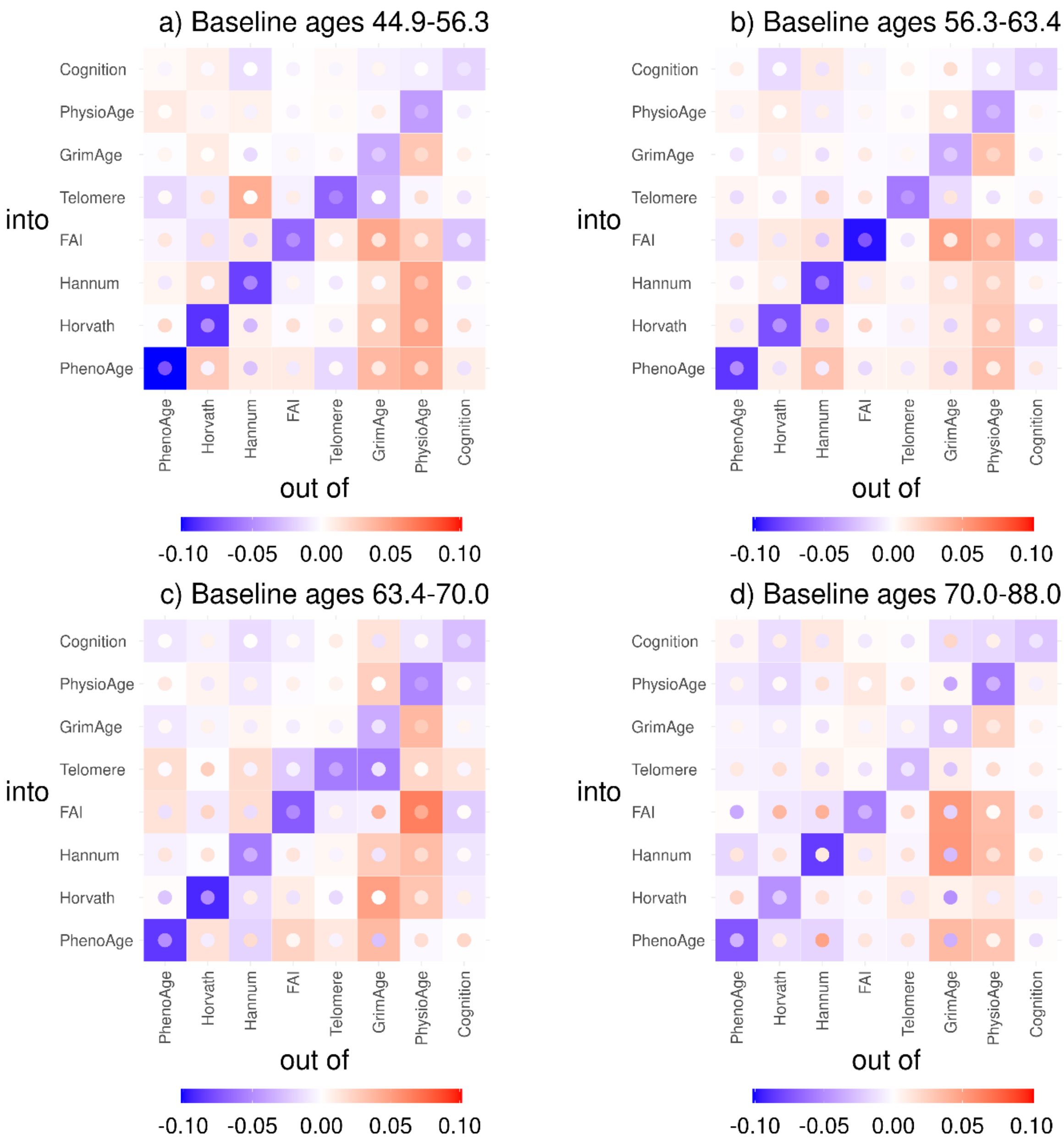

Figure S19: **Network estimates by age-cohort — main result.** We see that across ages, the dominant contributions of outgoing links are from PhysioAge and GrimAge. There are clear differences in sparsity across ages but there is no clear trend. The oldest group, **d**, appears to have a weaker diagonal than the other age ranges. This could be an indication of a loss of resilience or a subtle bias in the imputation model. Inner point is limit of 95% CI closest to 0: point is most visible for the least significant tiles; if point is opposite colour to tile then element is not significant.

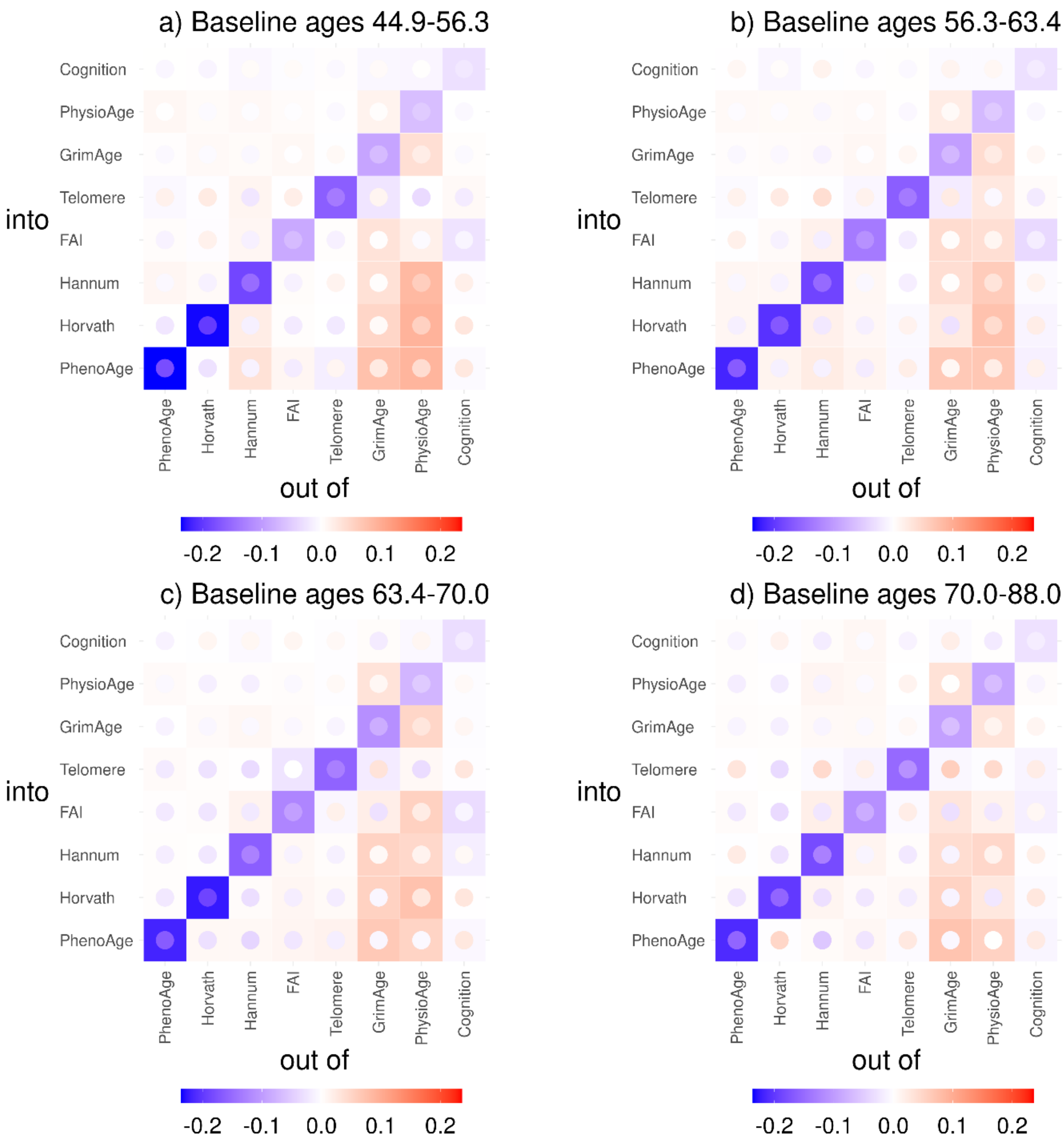

Figure S20: **Network estimates by age-cohort — MICE.** In the present figure we see strong similarity across ages whereas we did not in the main imputation method (Figure S19). Observe that the young network (**a**) coincides well with the main imputation method whereas the older individuals show visually lower agreement. This is an indication that MICE may rely too heavily on young individuals — whom are measured the most often — for training its imputation models, causing it to make older individuals look more like young individuals. This point is discussed in detail in Missing Data. Inner point is limit of 95% CI closest to 0: point is most visible for the least significant tiles; if point is opposite colour to tile then element is not significant.

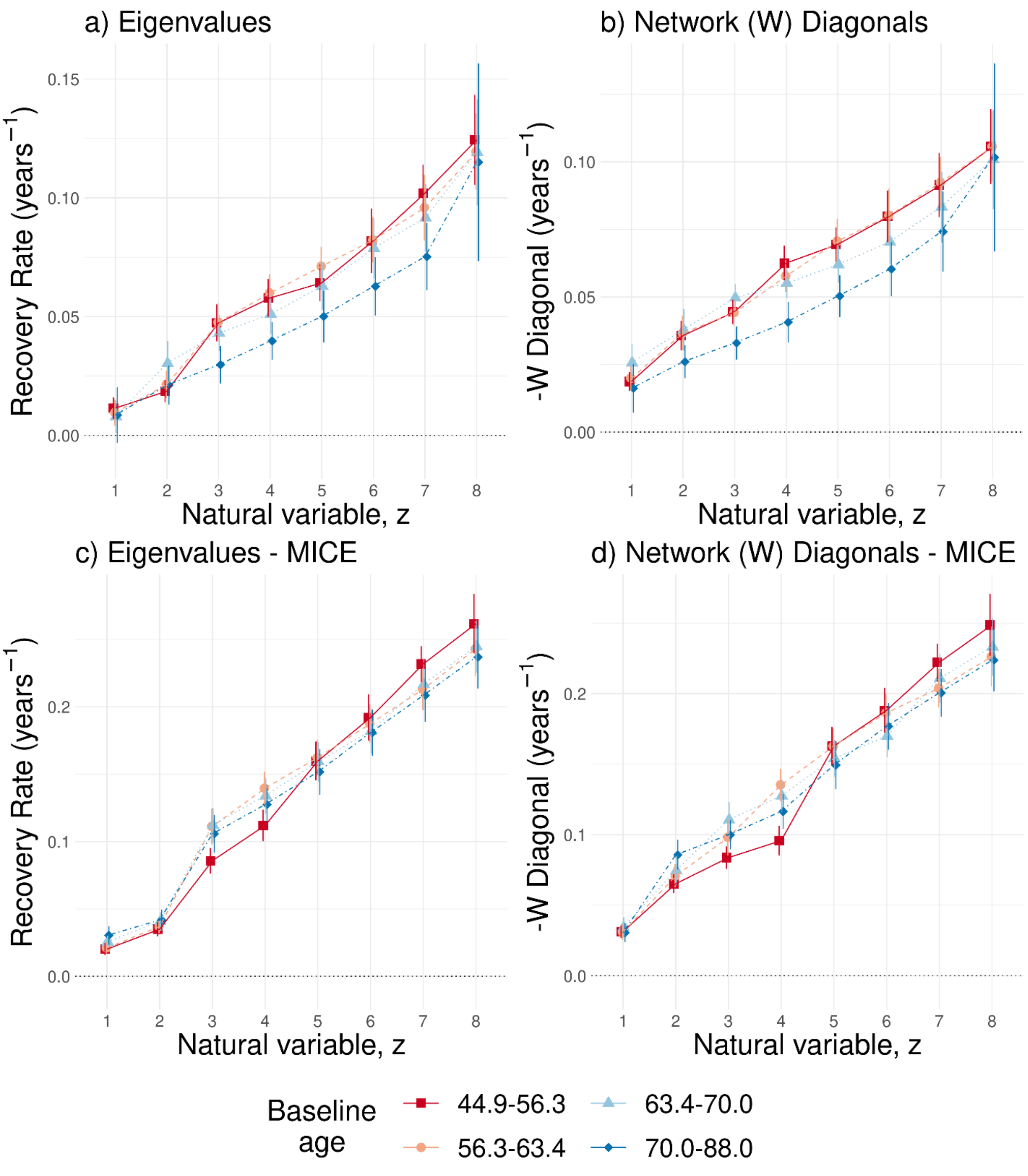

Figure S21: **Age-dependent stability**. Using the main imputation method (**a-b**) or MICE (**c-d**). There may be a trend of increasing or decreasing stability with age but it is typically small relative to the error bars. The most salient feature is the weak diagonal and associated eigenvalues for the oldest group, ages 70-88 in (**a-b**). We are skeptical that this is a real signal and not simply a consequence of the high levels of missingness in this group. In particular, the MICE imputed network does not show any weakening of the diagonal or eigenvalues (**c-d**). If we look at the overall networks we also see that the main imputation method seems to underestimate

the diagonal elements of $\boldsymbol{W}$ versus either MICE or available case (Figure S7). Error bars are from bootstrap, 100 resamples; (c-d) also includes error estimate of imputation.

Chronological Age and the Frailty Index (FI)

The dataset from Li *et al.*[3] includes longitudinal measurements of the FI. In the main analysis we withheld the FI as a proxy for longitudinal changes in health. The FI can be alternatively treated as yet another BA, with the caveat that it should be transformed to prevent issues with fitting (the model assumes normal errors, so it won't fit to FI=0 since it is always assuming symmetric errors above and below the mean). We transformed the FI using the lefthand side of Eq. (S5) with $\gamma/\alpha = 0.065$ and then scaled to match the mean and standard deviations of CA, specifically we multiplied by 22.11 then added 111.2. After transformation, the FI can be treated as normal along with the other BAs. For fitting, we pre-processed the input (BAs and CA) using PCA, picking the optimal number to minimize the 632-RMSE, which was 9 PCs (max: 10). The estimated network is presented in Figure S22. Where we have additionally included CA (age). The main features of the network are unchanged: PhysioAge still plays a central role with a secondary role for GrimAge (i.e. many outgoing links). Age (CA) appears to also have a central role, ostensibly representing unaccounted aging-related degrees-of-freedom. Note that the FI appears to be weakly connected to the others, primarily interacting with FAI. The FAI captures very similar phenomenon to the FI, in particular the FAI is composed of measures which were either directly included in the FI (self-reported hearing and vision) or which modify FI variables (e.g. ADLs)[8,9].

We can use the network to see how information propagates into the FI, a proxy for organism-level health. For example FAI, PhysioAge and Age can directly influence the FI and also gate all incoming information into the FI, whereas e.g. GrimAge must first modify one of those three to affect the FI. The implication is that genetic and epigenetic changes do not directly affect the FI. This is consistent with a recent longitudinal bivariate association study of the FI which found no association with Hannum, Horvath, PhenoAge or GrimAge, although they did find a directed association from DunedinPACE to the FI[23] (DunedinPACE is not included in the present study dataset).

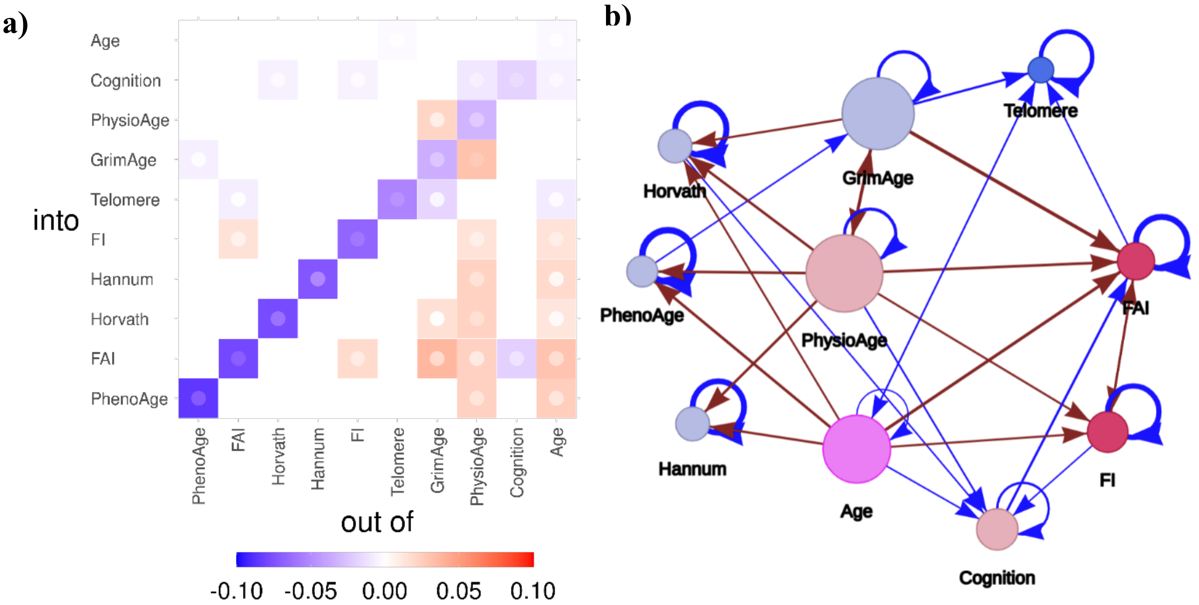

Figure S22: **Network interactome including FI and Age (chronological age)**. **a) Network weight matrix, W. b) Network representation.** Age and the FI can be included in the network, allowing us to see how information moves in and out. Observe that the essential features of the network are similar to the main text (Figure 1): with central nodes of PhysioAge and GrimAge. Age is another central node, ostensibly representing unaccounted aging effects that are not captured by the BAs. The FI appears to only connect with higher-level scales: PhysioAge, FAI and Age, having only indirect connections to epigenetic and genetic scales. The FI has been log-scale as described in the text. Both representations **a)** and **b)** are equivalent.

Works Cited

1. Mitnitski A, Rockwood K. Aging as a process of deficit accumulation: its utility and origin. *Interdiscip Top Gerontol*. 2015;40:85-98. doi:10.1159/000364933

2. Berglund K, Reynolds CA, Ploner A, et al. Longitudinal decline of leukocyte telomere length in old age and the association with sex and genetic risk. *Aging*. 2016;8(7):1398-1415. doi:10.18632/aging.100995

3. Li X, Ploner A, Wang Y, et al. Longitudinal trajectories, correlations and mortality associations of nine biological ages across 20-years follow-up. *Elife*. 2020;9. doi:10.7554/eLife.51507

4. Lu AT, Quach A, Wilson JG, et al. DNA methylation GrimAge strongly predicts lifespan and healthspan. *Aging*. 2019;11(2):303-327. doi:10.18632/aging.101684

5. Levine ME, Lu AT, Quach A, et al. An epigenetic biomarker of aging for lifespan and healthspan. *Aging*. 2018;10(4):573-591. doi:10.18632/aging.101414

6. Klemera P, Doubal S. A new approach to the concept and computation of biological age. *Mech Ageing Dev*. 2006;127(3):240-248. doi:10.1016/j.mad.2005.10.004

7. Reynolds CA, Finkel D, McArdle JJ, Gatz M, Berg S, Pedersen NL. Quantitative genetic analysis of latent growth curve models of cognitive abilities in adulthood. *Dev Psychol*. 2005;41(1):3-16. doi:10.1037/0012-1649.41.1.3

8. Finkel D, Sternäng O, Jylhävä J, Bai G, Pedersen NL. Functional Aging Index Complements Frailty in Prediction of Entry Into Care and Mortality. *J Gerontol A Biol Sci Med Sci*. 2019;74(12):1980-1986. doi:10.1093/gerona/glz155

9. Jiang M, Foebel AD, Kuja-Halkola R, et al. Frailty index as a predictor of all-cause and cause-specific mortality in a Swedish population-based cohort. *Aging*. 2017;9(12):2629-2646. doi:10.18632/aging.101352

10. Edemekong PF, Bomgaars DL, Sukumaran S, Levy SB. *Activities of Daily Living*. StatPearls Publishing, Treasure Island (FL); 2021. https://www.ncbi.nlm.nih.gov/books/NBK470404

11. Petersen, Kaare, Brandt and Pedersen, Michael, Syskind. The matrix cookbook. Published online 2012. https://www.math.uwaterloo.ca/~hwolkowi/matrixcookbook.pdf

12. Pridham G, Rutenberg AD. Network dynamical stability analysis reveals key “mallostatic” natural variables that erode homeostasis and drive age-related decline of health. *Sci Rep*. 2023;13(1):1-12. doi:10.1038/s41598-023-49129-7

13. Wood SN. Fast stable restricted maximum likelihood and marginal likelihood estimation of semiparametric generalized linear models. *J R Stat Soc Series B Stat Methodol*. 2011;73(1):3-36. doi:10.1111/j.1467-9868.2010.00749.x

14. White IR, Royston P, Wood AM. Multiple imputation using chained equations: Issues and guidance for practice. *Stat Med*. 2011;30(4):377-399. doi:10.1002/sim.4067

15. van Buuren S. *Flexible Imputation of Missing Data, Second Edition*. CRC Press; 2018. https://play.google.com/store/books/details?id=lzb3DwAAQBAJ

16. van Buuren S, Groothuis-Oudshoorn K. mice: Multivariate imputation by chained equations in R. *J Stat Softw*. 2010;45(3):1-68. https://dspace.library.uu.nl/handle/1874/44635

17. Hastie T, Tibshirani R, Friedman J. *The Elements of Statistical Learning: Data Mining, Inference, and Prediction*. Vol 2nd. Springer; 2017.

18. Robin X, Turck N, Hainard A, et al. pROC: an open-source package for R and S+ to analyze and compare ROC curves. *BMC Bioinformatics*. 2011;12:77. doi:10.1186/1471-2105-12-77

19. Mair W, Goymer P, Pletcher SD, Partridge L. Demography of dietary restriction and death in Drosophila. *Science*. 2003;301(5640):1731-1733. doi:10.1126/science.1086016

20. Cheetham NJ, Penfold R, Giunchiglia V, et al. The effects of COVID-19 on cognitive performance in a community-based cohort: a COVID symptom study biobank prospective cohort study. *eClinicalMedicine*. Published online July 21, 2023. doi:10.1016/j.eclinm.2023.102086

21. Pyrkov TV, Avchaciov K, Tarkhov AE, Menshikov LI, Gudkov AV, Fedichev PO. Longitudinal analysis of blood markers reveals progressive loss of resilience and predicts human lifespan limit. *Nat Commun*. 2021;12(1):2765. doi:10.1038/s41467-021-23014-1

22. Tarkhov AE, Denisov KA, Fedichev PO. Aging clocks, entropy, and the limits of age-reversal. *bioRxiv*. Published online October 11, 2022:2022.02.06.479300. doi:10.1101/2022.02.06.479300

23. Mak JKL, Karlsson IK, Tang B, et al. Temporal dynamics of epigenetic aging and frailty from midlife to old age. *J Gerontol A Biol Sci Med Sci*. Published online October 27, 2023. doi:10.1093/gerona/glad251
